# Analytical validation (accuracy, reproducibility, limit of detection) and gene expression analysis of FoundationOneRNA assay for fusion detection in 189 clinical tumor specimens

**DOI:** 10.1371/journal.pone.0329697

**Published:** 2025-09-12

**Authors:** Daokun Sun, Richard S. P. Huang, Michelle Green, Chenming Cui, Saumya D. Sisoudiya, Andrej Savol, Chang Xu, Cui Guo, Joel Skoletsky, Justin M. Allen, Khaled Tolba, Varun Pattani, Alyssa Tarzia, Jennifer Whiting, Yanhua Tang, Lee A. Albacker, Christine Vietz, Michael Datto, Jie He, William Richard Jeck

**Affiliations:** 1 Foundation Medicine, Inc., Boston, Massachusetts, United States of America; 2 Department of Pathology, Duke University, Durham, North Carolina, United States of America; Kore University of Enna: Universita degli Studi di Enna ‘Kore’, ITALY

## Abstract

Targeted DNA-based comprehensive genomic profiling (CGP) to detect clinically significant alterations is increasingly becoming standard for patients with advanced or recurrent cancer. RNA-based sequencing, however, may improve performance of fusion detection. We developed a robust targeted RNA sequencing assay (FoundationOne®RNA) and evaluated its analytic performance. FoundationOne®RNA is a hybrid-capture based targeted RNA sequencing test designed to optimally detect fusions (318 genes) and measure gene expression (1521 genes). Analytical validation studies were performed in College of American Pathologists (CAP)-accredited and Clinical Laboratory Improvement Amendments (CLIA)-certified lab to assess fusion call accuracy, assay reproducibility, limit of detection (LoD) and gene expression in 189 clinical solid tumor specimens. In the accuracy study, 160 out of 189 biopsy samples which were previously profiled using large-panel DNA- or RNA-based next-generation sequencing (NGS) passed quality control metrics and were studied using the FoundationOne®RNA assay. Analysis of all diagnostic fusions showed a positive percent agreement (PPA) of 98.28%, as well as a negative percent agreement (NPA) of 99.89% when compared to orthogonal assays. The FoundationOne®RNA assay was able to identify a low level *BRAF* fusion missed by orthogonal whole transcriptome RNA sequencing and was confirmed by fluorescence *in situ* hybridization (FISH). The range for the minimum RNA input and LoD was determined based on dilutions from 5 fusion-positive cell lines. It spans from 1.5ng (0.5% input) to 30ng (10% input) for RNA input and from 21 to 85 supporting reads for LoD. In the precision study, 10 out of 10 pre-defined target fusions had 100% reproducibility. In our gene expression analysis, multiple gene expression signatures were detected in fusion positive samples. FoundationOne®RNA assay successfully detected oncogenic fusions with high concordance to orthogonal NGS based tests, high reproducibility, and low limit of detection. This study demonstrated the robustness of FoundationOne®RNA and supports its use as a supplement to tissue DNA comprehensive genomic profiling (CGP) in routine clinical practice. Additional work is required to clarify optimal clinical scenarios for fusion detection and enable gene expression biomarkers for clinical use.

## Introduction

Precision oncology has revolutionized cancer treatment by tailoring therapies to the individual genomic alterations present in a patient’s tumor [1–7]. For example, several fusion genes (*ALK, ROS1, RET*, and *NTRK*) are currently recommended actionable molecular biomarkers for non-small cell lung cancer (NSCLC) by the American Society of Clinical Oncology (ASCO) guidelines [8]. In this paradigm, DNA-based CGP has emerged as the cornerstone for identifying actionable genomic aberrations and guiding treatment decisions [2,9]. However, detection of gene fusions with DNA-based CGP has certain limitations. While a well-designed DNA-based CGP assay has high sensitivity and specificity in detecting fusions, large, repetitive intronic regions make it difficult to achieve adequate coverage [10,11]. RNA is an attractive supplemental analyte to DNA-based CGP fusion detection because splicing mediated elimination of intronic regions in mRNA alleviates the need to sequence large intronic regions [10–12]. However, DNA remains the backbone for CGP due to the stability of DNA as compared to RNA and the ability to combine clinically relevant fusion detection with detection of single nucleotide variants, short indels and copy number alterations [13].

Testing RNA, by contrast, offers the ability to concurrently measure RNA expression of thousands of genes [14]. Importantly, gene expression may serve as a surrogate for immunohistochemistry (IHC). This could be leveraged both for diagnosis and predictive biomarker testing. This latter possibility to simultaneously assess RNA expression of thousands of genes may be increasingly important as antibody drug conjugates (ADC) with IHC biomarkers diversify [15,16]. Furthermore, gene expression reporting can reveal expression signatures by identifying the genes that exhibit consistent and significant changes in expression. While most pathogenic genomic DNA alterations typically lead to altered cell functions that drive oncogenesis, post-transcriptional modifications have the potential to alter the consequences of these DNA genomic alterations [17,18]. Unique patterns of RNA gene expression offered additional opportunity to characterize histologic subtypes or signal pathway activities as novel diagnosis and predictive biomarkers [19–21].

The utilization of RNA in addition to DNA in CGP represents an exciting avenue for refining precision oncology approaches. Not only can it enhance detection of currently clinically actionable fusions, but gene expression adds another data point to describe the cancer’s biology and holds the promise of more precise treatment for oncology patients [19–21]. Here, we examine a robust RNA-based CGP assay (FoundationOne®RNA) and present its analytical performance. The assay successfully detected known gene fusions with high sensitivity, specificity, reproducibility, and low limit of detection in a diverse set of cancer samples. We additionally examined the ability of FoundationOne®RNA to quantify gene expression levels and identify gene expression signatures, providing valuable insights into gene expression utility in clinical practice.

## Method

### Samples and materials

Among 189 samples evaluated in accuracy study, a total of 105 FFPE tissues were used for evaluating the concordance of clinically relevant fusion detection between FoundationOne®RNA and external orthogonal assays. 99 of the 105 were patient samples provided by Duke University Health System (DUHS) and the other 6 samples were procured from a commercial vendor. For samples provided by DUHS, previously generated orthogonal testing data was provided to compare with FoundationOne®RNA results. DUHS clinical samples and data were all collected as part of clinical standard of care and provided to the study through the Biorepository and Precision Pathology Center acting as the honest broker under IRB approval with waiver of consent (IRB# Pro00107750). Institutional Review Board (IRB) approval was obtained from the WIRB-Copernicus Group (WCG®) IRB prior to use of samples in the described validation studies. Foundation Medicine centrally reviewed H&E slides in each case for tumor content as part of standard operating procedure. For the 6 procured samples, orthogonal testing data was generated by sequencing extracted RNA with FoundationOne®Heme, a lab developed CGP assay. In addition to the FFPE cohort, 84 extracted RNA residual samples from routine clinical testing of FoundationOne® Heme were utilized to evaluate the concordance between FoundationOne®RNA and FoundationOne®Heme as previously analytically validated orthogonal test. This study is approved under IRB # 120160955 titled as “Use of Leftover Nucleic Acid samples that are not individually identifiable for analytical validation studies”. In precision study, ten FFPE samples harboring 10 fusions were processed on 3 different days, with 3 replicates per day, for a total of 9 replicates per source sample. All replicates (9 replicates X 10 source sample) passed quality control steps and were valid for reproducibility and repeatability analysis. In LoD study, five cell lines with known fusions were used to establish the LoD for FoundationOne®RNA. Extracted RNA from fusion-positive cell lines were pooled and titrated to five dilution levels with 10–20 replicates based on LoD study design. For accuracy and precision study, 500 ng RNA were inputted for each reaction. For LoD study, 300 ng RNA was used as input for each reaction. Sample selection strove to cover a range of tumor types and to replicate challenging conditions, such as samples close to the minimum tumor requirements (~20% tumor purity) and samples from tissues that are considered challenging to extract such as lung and prostate. FoundationOne®RNA assay analytical validation studies were executed between April 14^th^, 2021 and June 10^th^, 2021. The data were accessed on July 5^th^, 2021 for the evaluation purpose of this manuscript. Authors had no access to information that could identify individual participants during or after data collection.

### Description of workflow

**Fig 1:**
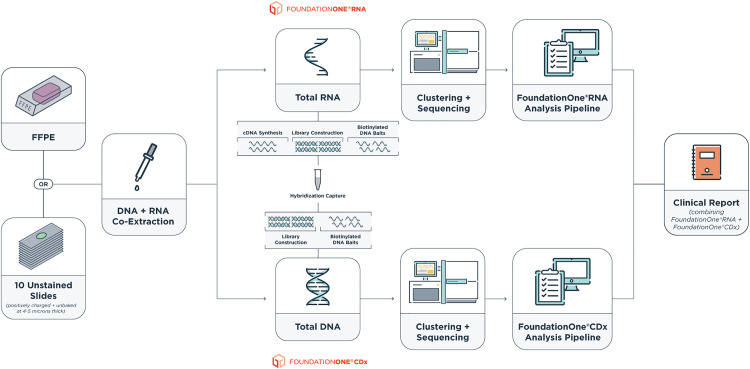
The integrated workflow of FoundationOne^®^CDx and FoundationOne^®^RNA assay. Both DNA and RNA were simultaneously extracted from FFPE samples or slides. RNA underwent sequencing and analysis using the FoundationOne^®^RNA pipeline, and it is highly recommended to include DNA sequencing by FoundationOne^®^CDx to generate a comprehensive clinical report.

FoundationOne®RNA is an NGS-based assay that was designed to optimally detect fusions (318 genes) and measure gene expression levels (1521 genes) (listed in Table S1 in S1 Appendix). FoundationOne®RNA is performed exclusively by Foundation Medicine as a central laboratory testing service using RNA extracted from tumor samples from cancer patients. The laboratory components are performed within a CAP-accredited and CLIA-certified laboratory that has been certified by Centers for Medicare & Medicaid Services (CMS) pursuant to CMS procedures for the certification of international laboratories in accordance with CLIA. DNA and RNA were co-extracted from FFPE samples or clinical slides through Foundation Medicine’s standard workflow [21]. RNA underwent sequencing and analysis using the FoundationOne®RNA pipeline, and DNA underwent sequencing and analysis through the FoundationOne®CDx [22] pipeline to generate a comprehensive clinical report (Fig 1). Process match controls were performed together with patient samples from library construction to sequencing. The process match controls are being assessed for reagent stability and overall workflow quality control metrics.

### Sequencing

The chosen libraries undergo pooling and sequencing using the Illumina HiSeq4000 platform, resulting in 30 million total sequence read pairs on average and over 3 million on-target distinct read pairs for each RNA sample.

### Rearrangement calling

A customized and proprietary alignment workflow was created to detect fusions from RNA sequencing data. Initially, raw sequence reads were aligned to the whole transcriptome (refSeq), and reads with suboptimal mapping were then aligned to whole genome references. The alignments to the reference genome were merged and calibrated using the complete genome reference (hg19) to enable fusion detection.

To identify gene rearrangements, clusters of chimeric read pairs were identified from RNA sequencing. In RNA, pairs mapping to refSeq sequences corresponding to different genes or to genomic loci more than 200 kbp apart were examined. The chimeric read clusters were filtered based on repetitive sequence content (with an average mapq score greater than 30) and the distribution of mapped positions (with a standard deviation greater than 10). The identified rearrangements were annotated based on the genomic loci of the clusters and categorized as gene fusions (e.g., EML4-ALK), gene rearrangements (e.g., *IGH-BCL2*), or truncating events (e.g., *TP53-TP53*). Rearrangement candidates were further filtered based on the number of chimeric reads supporting the events. For documented fusions, a minimum of 10 chimeric reads was required, while putative somatic driver rearrangements needed at least 50 chimeric reads.

In addition to the aforementioned *de novo* rearrangement detection method, reads were also aligned separately to a custom reference library specifically generated for common fusions and rearrangements. The generation of custom reference was blind to the fusions detected in this validation study. Fusions were identified by observing reads aligned across the junctions of rearrangement breakpoints.

### Gene expression analysis

In addition to variant detection among clinically relevant rearrangement genes, FoundationOne®RNA can also provide RUO reporting of gene expression quantitation values for an expanded set of cancer research relevant genes. Expression for genes with reliable quantitation is reported using TPM (transcripts per million) units, enabling inter-sample or inter-gene expression comparisons among cohorts of samples. All ten samples with replicates (9 replicates X 10 source sample) from precision study were examined in gene expression analysis. The statistical significance of expression between the rearrangement positive cohort and rearrangement negative cohort for selected genes was determined by a 1-sided Wilcoxon-Mann-Whitney rank-sum test.

Comparison of IHC-based ER/PR status with RNA gene expression-based measurement of *ESR1*/*PGR* genes was performed in 159 patients with breast cancer who received RNA-based profiling using FoundationOne®RNA during routine clinical care between June 2024 through September 2024. The samples and data were collected under IRB approval (IRB#20152817) with waiver of informed consent. The IHC-based ER/PR annotation was obtained from pathology reports of these patients. ER status (positive or negative) was available for all 159 patients whereas PR status (positive or negative) was available for 152 patients. TPM values of *ESR1* and *PGR* genes were available for all 159 patients.

### Concordance analysis

Let |M| be the total number of unique fusions reported by either FoundationOne®RNA or orthogonal tests in this study. Let *X*_*ij*_ be the detection status (*X*_*ij*_ = 1 for positive, 0 for negative) of the *i*^*th*^ variant in the *j*^*th*^ sample with the orthogonal assay, where i=1, 2, ⋯, |M| and j=1, 2,⋯, N. Similarly, let *Y*_*ij*_ be the detection status (*Y*_*ij*_ = 1 for positive, 0 for negative) of the *i*^*th*^ variant in the *j*^*th*^ sample with the FoundationOne®RNA assay, where i=1, 2, ⋯, |M| and j=1, 2,⋯, N.

The PPA, NPA, Positive predictive value (PPV), Negative predictive value (NPV) and Overall percent accuracy (OPA) were defined as:


$$PPA=\frac{\sum_{j=1}^{N}\sum_{i=1}^{\left|M\right|}X_{ij}\times Y_{ij}}{\sum_{j=1}^{N}\sum_{i=1}^{\left|M\right|}X_{ij}}\times 100\%$$

$$\;NPA=\frac{\sum_{j=1}^{N}\sum_{i=1}^{\left|M\right|}{(1-X}_{ij})\times {(1-Y}_{ij})}{\sum_{j=1}^{N}\sum_{i=1}^{\left|M\right|}{(1-X}_{ij})}\times 100\%$$

$$PPV=\frac{\sum_{j=1}^{N}\sum_{i=1}^{\left|M\right|}X_{ij}\times Y_{ij}}{\sum_{j=1}^{N}\sum_{i=1}^{\left|M\right|}Y_{ij}}\times 100\%$$

$$NPV=\frac{\sum_{j=1}^{N}\sum_{i=1}^{\left|M\right|}{(1-X}_{ij})\times {(1-Y}_{ij})}{\sum_{j=1}^{N}\sum_{i=1}^{\left|M\right|}{(1-Y}_{ij})}\times 100\%$$

$$OPA=\frac{\sum_{j=1}^{N}\sum_{i=1}^{\left|M\right|}\left|X_{ij}+Y_{ij}-1\right|}{N\times |M|}\times 100\%$$

95% confidence interval was calculated for PPA, NPA, PPV, NPV and OPA using Wilson’s method.

### Precision analysis

For fusion *i* in sample *j*, let $\;V_{ij}^{dr}$ be the replicate status (1 for valid replicate and 0 for invalid) of the $r^{th}(r=1,\;2,\;or\;3)$ replicate tested at the $d^{th}\;(d=1,\;2,\;3)$ day, and $x_{ij}^{dr}$ denotes the detection outcome (1 for detected and 0 for undetected).

The inter-run precision was quantified by reproducibility and calculated as:


$${\text{reproducibility}}_{ij}=\frac{\sum_{d=1}^{3}\sum_{r=1}^{3}I\left(x_{ij}^{dr}=1\right)\times V_{ij}^{dr}}{\sum_{d=1}^{3}\sum_{r=1}^{3}V_{ij}^{dr}}$$

The intra-run precision was measured as repeatability and calculated as:


$${\text{repeatability}}_{ij}=\frac{\sum_{d=1}^{3}I\left(x_{ij}^{d1}=x_{ij}^{d2}=x_{ij}^{d3}\right)\times V_{ij}^{d1}\times V_{ij}^{d2}\times V_{ij}^{d3}}{\sum_{d=1}^{3}V_{ij}^{d1}\times V_{ij}^{d2}\times V_{ij}^{d3}}$$

### Limit of detection analysis

The limit of detection describes the lowest level at which an analyte (genomic variant) can be consistently detected [23]. For RNA fusions called by the FoundationOne®RNA, minimum RNA input was determined as the lowest RNA input which achieved at least 95% hit rate for each fusion (defined below). LoD was reported as the mean supporting reads at minimum RNA input for each detected fusion.

To estimate the LoD of each RNA fusion in each cell line sample, empirical hit rate was calculated at each dilution level. Empirical hit rate was computed as the number of replicates with positive RNA fusion calls divided by the total number of valid replicates at each dilution level. For a RNA fusion, let *n*_*i*_ be the number of valid replicates at the *i*^*th*^ dilution level and *x*_*ij*_ be the detection outcome (1 for detected and 0 for undetected) of the target RNA fusion in *i*^*th*^ dilution level and *j*^*th*^ replicate. The empirical hit rate at the *i*^*th*^ dilution level is:


$$\text{Hit }{\text{rate}}_{i}\;=\;\frac{\sum_{j=\text{1}}^{n_{i}}I(x_{ij}=\text{1})}{n_{i}}$$

Estimated hit rates per detected RNA fusion were reported at each dilution level.

## Results

### Concordance with orthogonal assay

Concordance analysis evaluated variant level agreement in 81 valid commercial FFPE and 79 valid RNA residual samples against CLIA-certified orthogonal assays (S1 Fig). All evaluated fusions were confirmed to be detectable by both FoundationOne®RNA and orthogonal assay. Among these 160 samples from 32 different tissue types, we first evaluated the agreement of 26 unique actionable fusions from 13 genes (Table S2 in S1 Appendix). The PPA was 88.10% (37/42), PPV was 97.37% (37/38), and the NPA was 99.98% (4117/4118) (Table 1). Next, we examined the concordance of 28 unique diagnostic fusions (Table S3 in S1 Appendix), The PPA was 98.28% (57/58), PPV was 91.94% (57/62), and the NPA was 99.89% (4417/4422) (Table 1). We also evaluated the concordance of all clinically relevant fusions (including actionable fusions, diagnostic fusions, and other clinically relevant fusions) reported by an orthogonal assay for different sample types. In clinical FFPE samples and RNA residual samples, the PPA was 88.57% (31/35) and 89.25% (83/93) and NPA was 99.95% (2070/2071) and 99.98% (67358/67373), respectively (Table S4 in S1 Appendix). The positive concordance performance of FFPE and RNA residual samples was not significantly different (chi-square test, p-value = 0.913). In addition, we performed concordance analysis based on orthogonal assay type for all fusions reported by orthogonal assays (Table S5 in S1 Appendix). The PPA was 84.62% (22/26) and 90.29% (93/103) and NPA was 100% (1066/1066) and 99.98% (68387/68403), when comparing FoundationOne®RNA with DNA-based and RNA-based orthogonal assays, respectively. The positive concordance performance of DNA-based assay and RNA-based assay was not significantly different (chi-square test, p-value = 0.4057). Furthermore, we performed concordance analysis stratified by sample tumor purity and found minimal differences in fusion detection accuracy across different tumor purity levels (Table S6 in S1 Appendix). Notably, FoundationOne®RNA assay was able to identify a low level *KIAA1549-BRAF* fusion (*KIAA1549*(ex1–16 NM_020910)-*BRAF*(ex9–18 NM_004333)) missed by orthogonal whole transcriptome RNA sequencing and was subsequently confirmed by FISH. The FISH dual color probes target both BRAF and KIAA1549 to specifically detect this known fusion. There are 16% cells (>10% calling threshold) exhibiting overlapping/colocalizing BRAF-KIAA1549 signals which leads to the positive call. The most common reason for discordant variants was that, though supporting reads were present, the total number of chimeric reads were below the assay reporting threshold (50 chimeric reads) for putative driver somatic fusion. The raw data of detection status of each fusion, detailed discordant analysis and QC failure reasons were recorded in Tab Accuracy in S1 Dataset. Overall, the results suggest strong positive and negative agreement for fusion detection between FoundationOne®RNA and CLIA-certified orthogonal assays across different sample types (FFPE or RNA residual), orthogonal assay technologies (DNA-based or RNA-based), and a wide range of fusions (both actionable and diagnostic).

**Table 1:** The concordance analysis result of genes with actionable fusion and diagnostic fusion pairs.

	Metric	PPA (%)	NPA (%)	PPV (%)	NPV (%)	OPA (%)
**Actionable Fusion**	Point Estimate	88.10	99.98	97.37	99.83	99.79
	Count	(37/42)	(4117/4118)	(37/38)	(4117/4122)	(4154/4160)
	95% CI	[75, 94.81]	[99.86, 100.00]	[86.51, 99.53]	[99.72, 99.95]	[99.69, 99.93]
**Diagnostic Fusion**	Point Estimate	98.28	99.89	91.94	99.98	99.87
	Count	(57/58)	(4417/4422)	(57/62)	(4417/4418)	(4474/4480)
	95% CI	[90.86, 99.69]	[99.74, 99.95]	[82.47, 96.51]	[99.87, 100.00]	[99.72, 99.94]

Positive Percent Agreement (PPA), Negative Percent Agreement (NPA), Positive Percent Value (PPV), Negative Percent Value (NPV) and Overall Percent Agreement (OPA) were estimated with 95% confidence interval (Wilson’s method) for 26 unique actionable fusions (from 13 genes) as well as 28 unique diagnostic fusions, respectively. The actionable fusions and diagnostic fusions were listed in Table S2 and S3 in S1 Appendix. Actionable fusions may be targeted by small molecule inhibitors or antibodies, whereas diagnostic fusions are characteristic of certain tumor types and may confirm or suggest a particular diagnosis.

### Assay precision in clinical samples

**Fig 2:**
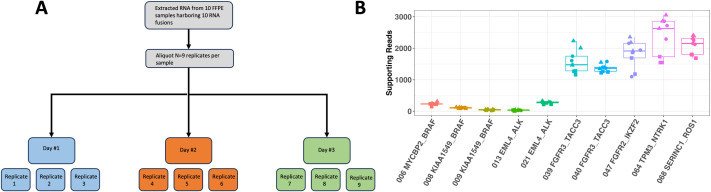
Precision study design and results of FoundationOne^®^RNA assay. **(A)** The experiment design of precision study. Ten Fusions from 10 valid commercial FFPEs were processed on 3 different days, with 3 replicates per day, for a total of 9 replicates per source sample. Equal cDNA input (500 ng) was used for each replicate. All replicates (9 replicates X 10 source sample) passed quality control steps and therefore were valid for reproducibility and repeatability analysis; **(B)** The distribution of supporting reads of 10 fusions. The supporting reads of 9 replicates were plotted for each fusion. Dots with the same shape were processed from the same day. First five fusions have low supporting reads and last 5 have high supporting reads. The prefix’FM_RNASeq_’ was omitted in sample names for brevity.

To assess the inter-run and intra-run precision (reproducibility and repeatability) of the FoundationOne®RNA assay, ten FFPE samples from 7 different cancer types harboring 10 clinically relevant fusions were selected and run repeatedly with the FoundationOne®RNA assay. Three replicates processed per run, and runs starting on 3 unique days for a total of 9 replicates per sample (3x3=9) (Fig 2A). All 10 fusions had 100% (9/9) reproducibility and 100% (3/3) repeatability (Table 2). We also examined the distribution of supporting reads of 10 fusions. The distribution of supporting reads was largely consistent across 9 replicates for each fusion (Fig 2B). Notably, the *EML4-ALK* fusion from FM_RNASeq_013 and *KIAA1549-BRAF* fusion from FM_RMASeq_009 (Fig 2B) have relatively low supporting reads across 9 replicates (Mean supporting read were 32 and 49, respectively), but FoundationOne®RNA assay still achieved 100% reproducibility and repeatability, which highlighted that it is capable of accurately and reproducibly detecting and characterizing fusions even in challenging cases with low RNA signals. The raw data of these 10 fusions used in precision study was provided in Tab Precision in S1 Dataset.

**Table 2:** The reproducibility and repeatability results of precision study.

Source Sample	Fusion	Alteration Subtype	Reproducibility (%)	Repeatability (%)
FM_RNASeq_006	MYCBP2-BRAF	fusion	100.00 (9/9)	100.00 (3/3)
FM_RNASeq_008	KIAA1549-BRAF	fusion	100.00 (9/9)	100.00 (3/3)
FM_RNASeq_009	KIAA1549-BRAF	fusion	100.00 (9/9)	100.00 (3/3)
FM_RNASeq_013	EML4-ALK	fusion	100.00 (9/9)	100.00 (3/3)
FM_RNASeq_021	EML4-ALK	fusion	100.00 (9/9)	100.00 (3/3)
FM_RNASeq_039	FGFR3-TACC3	fusion	100.00 (9/9)	100.00 (3/3)
FM_RNASeq_040	FGFR3-TACC3	fusion	100.00 (9/9)	100.00 (3/3)
FM_RNASeq_047	FGFR2-IKZF2	fusion	100.00 (9/9)	100.00 (3/3)
FM_RNASeq_064	TPM3-NTRK1	fusion	100.00 (9/9)	100.00 (3/3)
FM_RNASeq_068	SERINC1-ROS1	fusion	100.00 (9/9)	100.00 (3/3)
Overall	All fusions		100 (90/90) (95.91, 100)	100 (30/30) [88.65, 100]

Inter-run reproducibility and intra-run repeatability were evaluated for each fusion and all fusions combined. For all fusions combined, 95% confidence intervals were calculated for reproducibility and repeatability using Wilson’s method.

### Assay limit of detection in contrived samples

**Fig 3:**
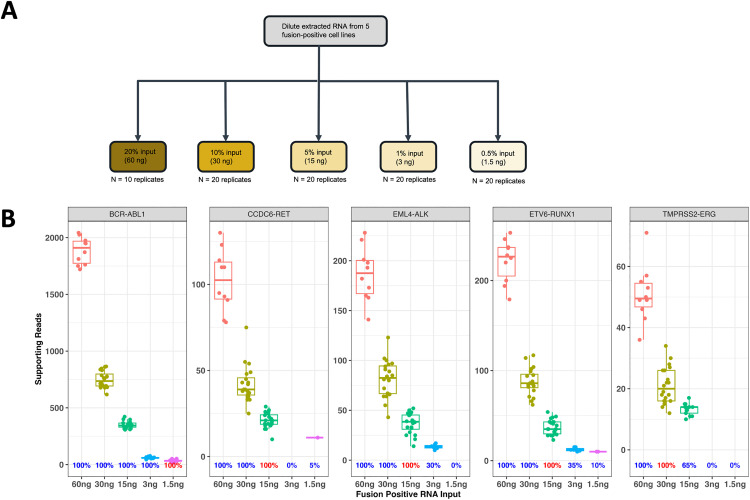
LoD study design and results of FoundationOne^®^RNA assay. **(A)** The experiment design of LoD study. Five known fusions from 5 fusion-positive cell lines were selected. Extracted RNA from fusion-positive cell lines were titrated to five dilution levels. The RNA from each cell line was pooled and diluted as needed. Fusion Percent input was based on 300ng cDNA synthesis reaction; **(B)** The boxplots of supporting reads per input RNA level for each of the five known fusion. The hit rate of each dilution level was annotated along x-axis and the hit rate of LoD was highlighted.

To assess the limit of detection (LoD) across various fusions, five fusion positive cell lines were examined with 5 dilution levels (Fig 3A). Five fusions were known variants in selected fusion positive cell lines and three were other fusions detected by FoundationOne®RNA in selected cell lines. Hit rates were calculated per fusion at each diluent level, summarized in Table 3 and Table S7 in S1 Appendix, and plotted in Fig 3B and S2 Fig for visualization. The number of supporting reads decreased with reduction of RNA input as expected. Minimum RNA input was determined as the lowest RNA input which achieved at least 95% hit rate for each fusion. LoD was reported as the mean supporting reads at minimum RNA input for each fusion. We analyzed LoD using supporting reads as a metric as it was the actual signal detected by the sequencer and could be a genuine estimate of LoD in the context of an NGS assay. For the five known RNA fusions, the minimum RNA input ranged from 1.5 ng (0.5% input) to 30 ng (10% input) and the LoD spanned from 21 to 37 supporting reads. For the three other fusions detected in fusion positive cell lines, the minimum RNA input ranged from 15 ng (5% input) to 30 ng (10% input) and the LoD spanned from 36 to 85 supporting reads. Notably, the RNA fusion *BCR-ABL1* had a 100% hit rate at all five levels, so the true minimum RNA input was under 1.5 ng (0.5% input) and the authentic LoD stood below 33 supporting reads. The variability of LoD of each fusion was also observed in FoundationOne® CDx and FoundationOne® Liquid CDx [22,24]. In total, our LoD study indicated that the FoundationOne®RNA assay is very sensitive and reliable in fusion detection in the setting of low total input RNA. The raw data of these 8 fusions evaluated in LoD study was provided in Tab LoD in S1 Dataset.

**Table 3:** The results of LoD study for five known fusions.

Cell Line	Fusion	Method	Minimum RNA Input	LoD	LoD Hit Rate
K562	BCR-ABL1	Hit Rate	1.5ng (0.5% input)	33	100% (20/20)
LC-2/ad	CCDC6-RET	Hit Rate	15ng (5% input)	21	100% (20/20)
NCI-H2228	EML4-ALK	Hit Rate	15ng (5% input)	37	100% (20/20)
Reh	ETV6-RUNX1	Hit Rate	15ng (5% input)	37	100% (20/20)
NCI-H660	TMPRSS2-ERG	Hit Rate	30ng (10% input)	21	100% (20/20)

Minimum RNA input was determined as the lowest RNA input which achieved at least 95% hit rate for each fusion. LoD was reported as the mean supporting reads at minimum RNA input for each fusion.

### Gene expression analysis

**Fig 4:**
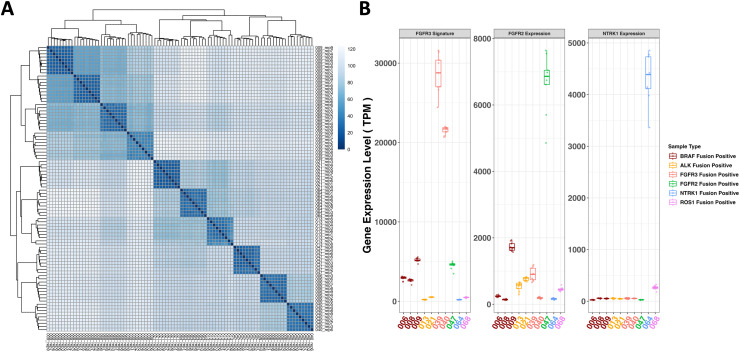
Gene expression analysis on FoundationOne^®^RNA. **(A)**Ten samples with 9 replicates each (x-axis and y-axis were in same identifier order) from precision study were used in this sample-to-sample distance plot. Darkest shade of blue corresponds to the minimum value (sample-sample distance calculated from Euclidean distance using log2(TPM) of all 1521 gene expression reporting genes) in the data, while the lightest shade of blue will represent the maximum value. The gradient used to represent the range of values in the distance matrix. All replicates were clustered in its sample-wide clade, which suggested remarkable gene expression reporting reproducibility that arrived the experimental design. The prefix ’FM_RNASeq_’ was omitted in row and column names for brevity; **(B)** The FGFR3 signature was determined by calculating the sum of expression (measured in TPM – Transcripts Per Million) of three genes: FGFR3, TP63, and WNT7B. FGFR2 and NTRK1 expression level was determined by calculating the expression (measured in TPM – Transcripts Per Million) of FGFR2 and NTRK1 gene, respectively. FGFR3 fusion positive samples (2 samples in red color with 9 replicates for each) indicated up-regulated differential gene expression levels (Wilcoxon rank sum test, fold change > 17, p < 0.001) compared to samples with other fusions (8 samples with 9 replicates for each). FGFR2 fusion positive sample (1 sample in green color with 9 replicates for each) indicated up-regulated differential gene expression level (Wilcoxon rank sum test, fold change > 16, p < 0.001) compared to samples with other fusions (9 samples with 9 replicates for each). NTRK1 fusion positive sample (1 sample in blue color with 9 replicates for each) indicated up-regulated differential gene expression level (Wilcoxon rank sum test, fold change > 83, p < 0.001) compared to the samples with other fusions (9 samples with 9 replicates for each). The prefix ’FM_RNASeq_’ was omitted in sample names for brevity.

Gene expression analysis was conducted on FoundationOne®RNA using 10 samples from precision study presented valuable insights into the samples’ expression signatures. The sample-to-sample distance plot (Fig 4A) utilized 10 samples, each with 9 replicates showed that all replicates clustered within their respective sample-wide clades and aligned with the experimental design, affirming the assay’s reproducibility in reporting gene expression. The *FGFR3* signature (Fig 4B), derived from the expression levels of three genes (*FGFR3, TP63*, and *WNT7B*) [25], demonstrated its potential application in fusion gene signature detection. Statistical analysis indicated that two *FGFR3* fusion positive samples, each with 9 replicates, exhibited significantly up-regulated differential gene expression levels (Wilcoxon rank sum test, fold change > 17, p < 0.001) compared to those 8 FGFR3 fusion negative samples. *FGFR2* and *NTRK1* expression level (Fig 4B) were determined by calculating the expression of *FGFR2* and *NTRK1* genes, respectively. One *FGFR2* fusion positive sample showed up-regulated differential gene expression level (Wilcoxon rank sum test, fold change > 16, p < 0.001) compared to the 9 FGFR2 fusion negative samples. One NTRK1 fusion positive sample was up-regulated in differential gene expression level (Wilcoxon rank sum test, fold change > 83, p < 0.001) compared to the 9 NTRK1 fusion negative samples. It was expected that the presence of *FGFR3*, *FGFR2* and *NTRK1* fusions resulted in gene overexpression, because they were all kinase-activating fusions which would trigger tyrosine kinase signaling cascade and downstream transcription machinery [26–28]. In addition, we examined the expression level of *ESR1* and *PGR*, stratified by ER and PR positivity based on IHC results, respectively. *ESR1* expression was significantly higher in ER+ cases (n = 104) compared to ER- cases (n = 55) (Wilcoxon rank sum test, fold change > 61, p < 0.001). Similarly, median expression of *PGR* gene was approximately 34-fold higher in PR+ cases (n = 65) compared to PR- cases (n = 87) (Wilcoxon rank sum test, fold change > 34, p < 0.001) (S3 Fig). The provided results demonstrate a strong correlation between *ESR1* and *PGR* RNA expression levels and their IHC status, indicating that RNA expression could serve as a reliable substitute for IHC. The comprehensive findings reinforce the assay’s reliability and consistent performance in detecting different gene expression patterns across various samples. The gene expression data was provided in S2 Dataset.

## Discussion

This study presents the analytic validation data for FoundationOne®RNA. The results of this study highlight the accuracy, reliability, and effectiveness of the FoundationOne®RNA assay in detecting oncogenic fusions, with a strong agreement observed when compared to orthogonal NGS based assays. Another notable finding from this study is the ability of the FoundationOne®RNA assay to maintain very high reproducibility, even in cases with limited fusion read support. This is a significant advantage, as it allows for the robust detection of clinically relevant fusions that might have been missed by other sequencing-based assays with stricter supporting reads requirements. In cases where fusions were not detected by this assay, the presence of chimeric reads below current thresholds suggests that performance may improve further as reporting thresholds are adjusted with algorithm updates, additional data and working experience with the assay.

The LoD study showed low minimum RNA input and supporting reads requirements for the FoundationOne®RNA assay. This demonstrates the sensitivity of the assay in detecting fusion events, even at low RNA input level. Importantly, accurate real-world detection of gene fusion for RNA based assays may vary with the gene fusion in question, due in no small part to variability in expression of the fusion product relative to other transcripts. For fusion products where the mechanism of oncogenicity is overexpression, the minimum RNA input may be lower than for non-overexpressed fusion gene products.

In the accuracy study, the age of the FFPE specimens and tumor contents were assessed as part of the study workflow and no obvious association was observed between the quality of specimens and analytical performance of fusion detection (Tab Accuracy in S1 Dataset). The ability to detect fusions at such low levels of input material and sequencing signal further solidifies the reliability and clinical utility of the FoundationOne®RNA assay.

In addition to detecting fusions, we also demonstrated the capability of the FoundationOne®RNA assay to feasibly detect gene expression signatures. The ability to detect expression for multiple genes at once could become increasingly important with the numerous emerging ADC with accompanying IHC biomarkers being developed, as RNA gene expression can potentially be utilized as a surrogate, replacement, or screening biomarker for IHC (S3 Fig). Additionally, RNA gene expression can be a more reproducible methodology when compared to IHC as assessment of *PDL-1* and *HER2* expression by IHC has revealed a concerning level of variability and poor reproducibility in clinical settings [29–32]. RNA gene expression signatures may also be utilized clinically in the future. For example, differential *FGFR3* expression has been associated with various malignancies, including bladder cancer and multiple myeloma, where overexpression or activating mutations in *FGFR3* contribute to tumor growth and progression [33–35]. Detecting an *FGFR3* expression signature in tumor samples can serve as a potential diagnostic and prognostic marker, guiding treatment decisions [36–38]. Understanding and monitoring *FGFR3* expression profiles can thus inform tailored therapeutic approaches and improve patient outcomes in these medical contexts.

In conclusion, this study highlights the robustness and reliability of the FoundationOne®RNA assay in detecting oncogenic fusions, even in cases with low levels of input material and supporting reads. Notably, the ability to co-extract DNA and RNA with no additional sample required when compared to DNA only CGP remains an important consideration for adding RNA on to DNA-based testing in this current trend of increasingly smaller tissue specimens available for tissue CGP testing. The gene expression reporting capabilities for research use and potential later clinical implementation further strengthens the case for incorporating FoundationOne®RNA as a supplement to tissue DNA-based CGP in routine clinical practice. Additional population level studies should be performed to quantify the number of fusions detected by RNA but not detected by DNA-based CGP in various tumor types. The findings of this study support the use of the FoundationOne®RNA assay as a valuable and accurate tool in precision oncology.

## Supporting information

S1 Fig The experiment design of accuracy study. Among 105 clinical FFPE samples and 84 clinical RNA residual samples, 24 and 5 had unsuccessful quality control steps, respectively (QC failure reason were listed in Tab Accuracy in S1 Dataset). The higher failure rate in clinical FFPE samples were likely due to biomolecule crosslinking, nucleic acid fragmentation and low RNA stability in FFPE blocks. Fusions from 81 valid commercial FFPE and 79 valid RNA residual samples were evaluated for concordance against orthogonal assays. Clinical FFPE samples were previously screened for RNA fusions using orthogonal testing. Clinical RNA residual samples were processed from clinical FoundationOne Heme residual RNA.
(DOCX)

S2 Fig The expanded result of LoD study. The boxplots of supporting reads per input RNA level for each fusion. The hit rate of each dilution level was annotated along x-axis and the hit rate of LoD was highlighted. First five fusions were known fusions in selected fusion positive cell lines and the following three were other fusions detected in fusion positive cell lines.
(DOCX)

S3 Fig *ESR1*/*PGR* RNA expression strongly correlates with IHC status. (A) *ESR1* gene expression (TPM) among ER+ (n = 104) vs ER- cases (n = 55). Median: 6,848 TPM [IQR: 2,189–10,789] in ER + vs. 111 TPM [IQR: 69–222] in ER-; (B) *PGR* gene expression (TPM) among PR+ (n = 65) vs PR- cases (n = 87). median: 241 TPM [IQR 43–748] in PR + vs. 7 TPM [IQR 3–12] in PR-. ER/PR status was determined by IHC. Statistics were determined using a Wilcoxon rank sum test.
(DOCX)

S1 Appendix Tables S1–S7. (DOCX)

S1 Dataset One variant per line data for analytical validation (Accuracy, Precision and LoD) study. (XLSX)

S2 Dataset Gene expression raw data. (CSV)
