# Supplementary material for: Analytical validation (accuracy, reproducibility, limit of detection) and gene expression analysis of FoundationOneRNA assay for fusion detection in 189 clinical tumor specimens
Source: PLoS One. 2025 Sep 12;20(9):e0329697. doi: 10.1371/journal.pone.0329697 (PMC12431237; doi:10.1371/journal.pone.0329697)
Supplement: S1 Fig — Among 105 clinical FFPE samples and 84 clinical RNA residual samples, 24 and 5 had unsuccessful quality control steps, respectively (QC failure reason were listed in Tab Accuracy in S1 Dataset). The higher failure rate in clinical FFPE samples were likely due to biomolecule crosslinking, nucleic acid fragmentation and low RNA stability in FFPE blocks. Fusions from 81 valid commercial FFPE and 79 valid RNA residual samples were evaluated for concordance against orthogonal assays. Clinical FFPE samples were previously screened for RNA fusions using orthogonal testing. Clinical RNA residual samples were processed from clinical FoundationOne Heme residual RNA. (DOCX) [file pone.0329697.s001.docx]

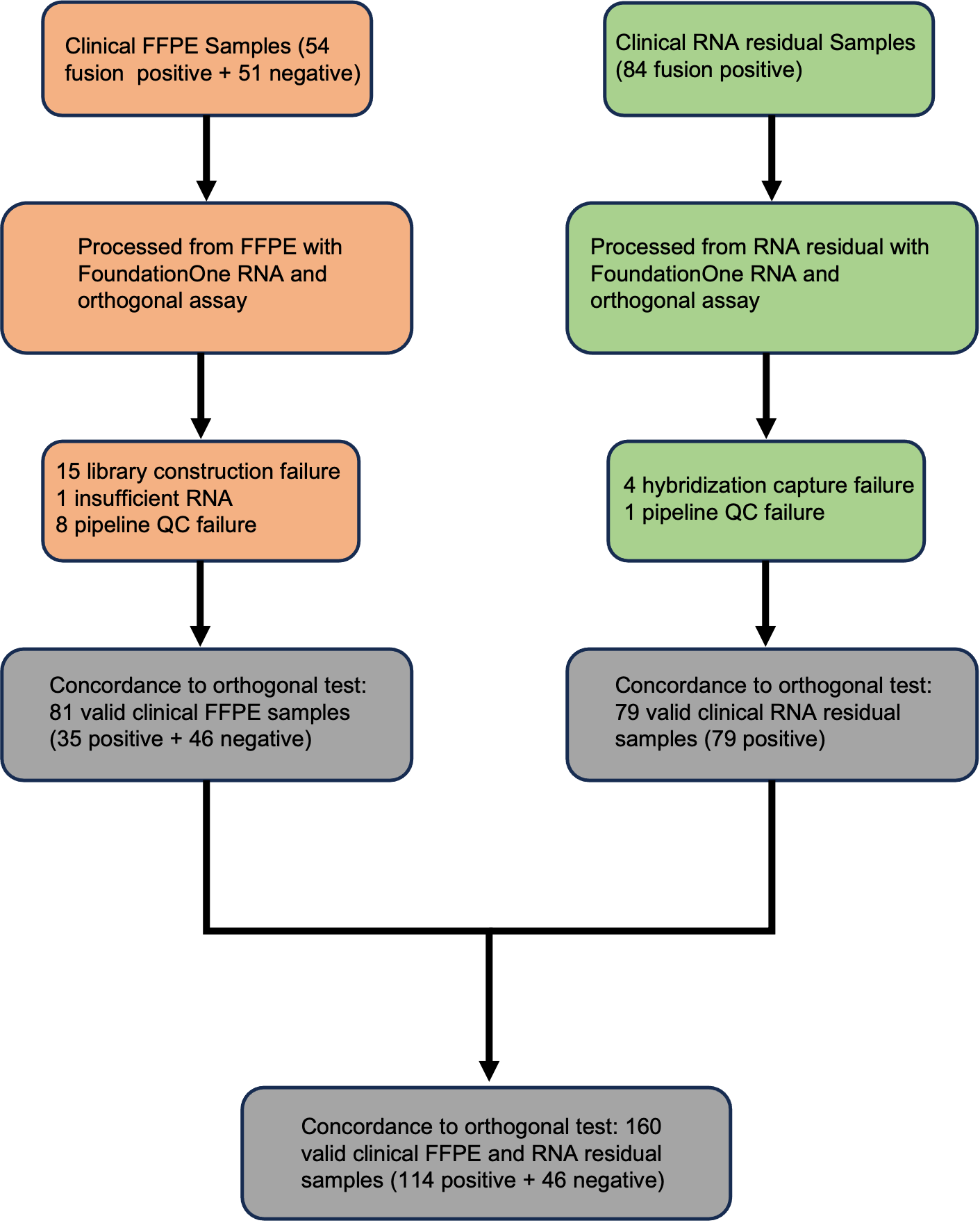


**S1 Fig. The experiment design of accuracy study.**

Among 105 clinical FFPE samples and 84 clinical RNA residual samples, 24 and 5 had unsuccessful quality control steps, respectively (QC failure reason were listed in **Tab Accuracy in S1 Dataset**). The higher failure rate in clinical FFPE samples were likely due to biomolecule crosslinking, nucleic acid fragmentation and low RNA stability in FFPE blocks. Fusions from 81 valid commercial FFPE and 79 valid RNA residual samples were evaluated for concordance against orthogonal assays. Clinical FFPE samples were previously screened for RNA fusions using orthogonal testing. Clinical RNA residual samples were processed from clinical FoundationOne Heme residual RNA.
