# Supplementary material for: Analytical validation (accuracy, reproducibility, limit of detection) and gene expression analysis of FoundationOneRNA assay for fusion detection in 189 clinical tumor specimens
Source: PLoS One. 2025 Sep 12;20(9):e0329697. doi: 10.1371/journal.pone.0329697 (PMC12431237; doi:10.1371/journal.pone.0329697)
Supplement: S2 Fig — The boxplots of supporting reads per input RNA level for each fusion. The hit rate of each dilution level was annotated along x-axis and the hit rate of LoD was highlighted. First five fusions were known fusions in selected fusion positive cell lines and the following three were other fusions detected in fusion positive cell lines. (DOCX) [file pone.0329697.s002.docx]

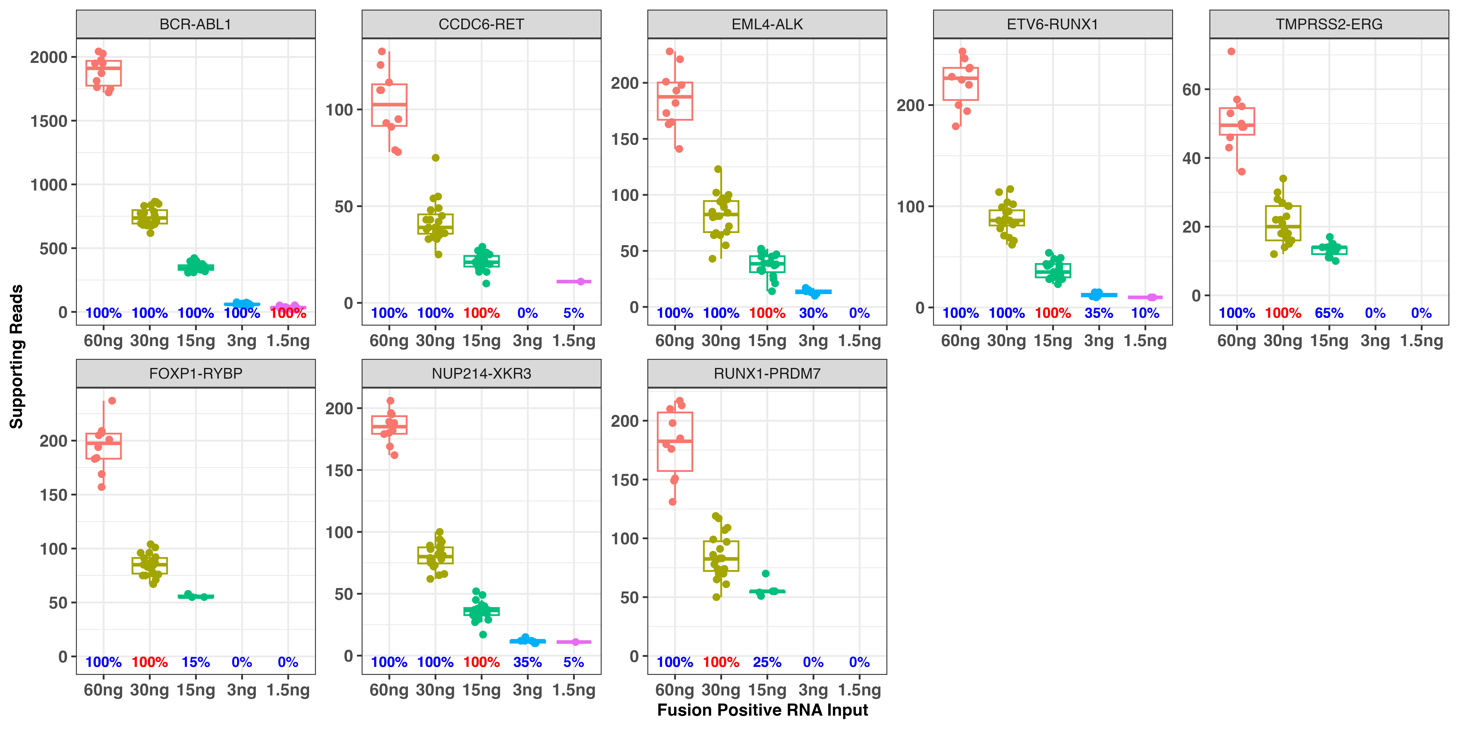


**S2 Fig. The boxplots of LoD study expanded result.**

The boxplots of supporting reads per input RNA level for each fusion. The hit rate of each dilution level was annotated along x-axis and the hit rate of LoD was highlighted. First five fusions were known fusions in selected fusion positive cell lines and the following three were other fusions detected in fusion positive cell lines.
