# Supplementary material for: Analytical validation (accuracy, reproducibility, limit of detection) and gene expression analysis of FoundationOneRNA assay for fusion detection in 189 clinical tumor specimens
Source: PLoS One. 2025 Sep 12;20(9):e0329697. doi: 10.1371/journal.pone.0329697 (PMC12431237; doi:10.1371/journal.pone.0329697)
Supplement: S3 Fig — (A) ESR1 gene expression (TPM) among ER+ (n = 104) vs ER- cases (n = 55). Median: 6,848 TPM [IQR: 2,189–10,789] in ER + vs. 111 TPM [IQR: 69–222] in ER-; (B) PGR gene expression (TPM) among PR+ (n = 65) vs PR- cases (n = 87). median: 241 TPM [IQR 43–748] in PR + vs. 7 TPM [IQR 3–12] in PR-. ER/PR status was determined by IHC. Statistics were determined using a Wilcoxon rank sum test. (DOCX) [file pone.0329697.s003.docx]

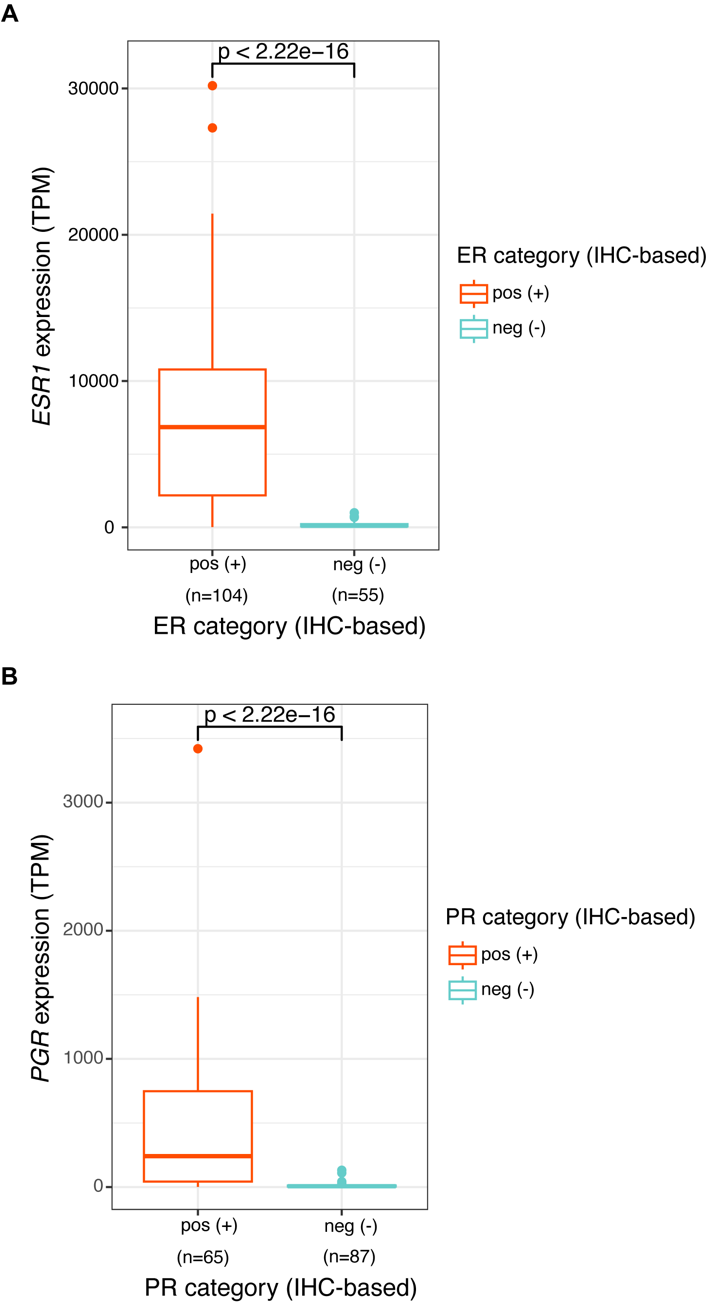


**S3 Fig. *ESR1*/*PGR* RNA expression strongly correlates with IHC status.**

(A) *ESR1* gene expression (TPM) among ER+ (n=104) vs ER- cases (n=55). Median: 6,848 TPM [IQR: 2,189–10,789] in ER+ vs. 111 TPM [IQR: 69–222] in ER-; (B) *PGR* gene expression (TPM) among PR+ (n=65) vs PR- cases (n=87). median: 241 TPM [IQR 43-748] in PR+ vs. 7 TPM [IQR 3-12] in PR-.

ER/PR status was determined by IHC. Statistics were determined using a Wilcoxon rank sum test.
