# Supplementary material for: Analytical validation (accuracy, reproducibility, limit of detection) and gene expression analysis of FoundationOneRNA assay for fusion detection in 189 clinical tumor specimens
Source: PLoS One. 2025 Sep 12;20(9):e0329697. doi: 10.1371/journal.pone.0329697 (PMC12431237; doi:10.1371/journal.pone.0329697)
Supplement: S1 Appendix — (DOCX) [file pone.0329697.s004.docx]

**S1 Supplemental Appendix: Table S1-S7**

**Table S1. Lists of detectable genes by FoundationOne®RNA**

| **Fusion Detection Genes** | **Gene Expression Genes** |
| --- | --- |
| ABCB1 | A2M |
| ABL1 | ABCA6 |
| ABL2 | ABCB1 |
| ACSL6 | ABCC2 |
| AFF1 | ABCC9 |
| AFF4 | ABI1 |
| AKT1 | ABL1 |
| AKT2 | ABL2 |
| AKT3 | ACACA |
| ALK | ACLY |
| APC | ACRBP |
| AR | ACSL3 |
| ARHGAP26 | ACSL6 |
| ARHGDIA | ACTA2 |
| ARID1A | ACTG1 |
| ARID1B | ACTG2 |
| ASXL1 | ACTN1 |
| ATIC | ACTR3B |
| ATM | ACVR1 |
| ATR | ACVR1C |
| AXL | ACVRL1 |
| B2M | ADAM12 |
| BAP1 | ADAM19 |
| BCL10 | ADAM2 |
| BCL11B | ADCY7 |
| BCL2 | ADGRB1 |
| BCL3 | ADGRB3 |
| BCL6 | ADGRF5 |
| BCOR | ADGRL4 |
| BCR | ADRB2 |
| BRAF | AFF1 |
| BRCA1 | AFF3 |
| BRCA2 | AFF4 |
| BRD3 | AFP |
| BRD4 | AGR2 |
| BRIP1 | AGR3 |
| BTG1 | AHR |
| BTK | AIFM3 |
| CAMTA1 | AKT1 |
| CBFA2T3 | AKT2 |
| CBFB | AKT3 |
| CBL | ALDH2 |
| CCND1 | ALK |
| CCND2 | ALOX12 |
| CCND3 | AMER1 |
| CCNE1 | AMZ1 |
| CD19 | ANGPT1 |
| CD274 | ANGPT2 |
| CD28 | ANLN |
| CD38 | ANPEP |
| CD74 | ANXA1 |
| CDH1 | ANXA2 |
| CDK4 | APC |
| CDK6 | APCDD1 |
| CDKN2A | APEX2 |
| CDKN2B | APH1A |
| CEBPA | APLN |
| CIC | APOBEC3A |
| CIITA | APOBEC3B |
| CLDN18 | APOBR |
| CLDN3 | APOL6 |
| CLTC | APP |
| CNTRL | APPBP2 |
| COL1A1 | AR |
| CREB3L1 | AREG |
| CREB3L2 | ARF1 |
| CREBBP | ARG2 |
| CRLF2 | ARHGAP15 |
| CSF1 | ARHGAP26 |
| CUX1 | ARHGDIA |
| CXCR4 | ARID1A |
| CYLD | ARID1B |
| DDIT3 | ARID3A |
| DDR1 | ARNT |
| DDR2 | ARNT2 |
| DDX10 | ASAP2 |
| DEK | ASB13 |
| DHH | ASCL2 |
| DIRC2 | ASGR2 |
| DLC1 | ASTE1 |
| DNMT3A | ASXL1 |
| DUSP22 | ATAD2 |
| EBF1 | ATIC |
| EGFR | ATM |
| EIF4A2 | ATP2C1 |
| ELF4 | ATP8A1 |
| ELL | ATP8B2 |
| ELN | ATR |
| EML4 | AURKA |
| EP300 | AURKB |
| EPOR | AVPR1A |
| EPS15 | AXIN2 |
| ERBB2 | AXL |
| ERBB3 | B2M |
| ERG | B3GNT5 |
| ESR1 | BAALC |
| ETS1 | BAALC-AS2 |
| ETV1 | BAG1 |
| ETV4 | BAG2 |
| ETV5 | BAGE4 |
| ETV6 | BAK1 |
| EWSR1 | BAMBI |
| FBXW7 | BAP1 |
| FEV | BASP1 |
| FGFR1 | BATF |
| FGFR1OP | BATF3 |
| FGFR2 | BAX |
| FGFR3 | BAZ2B |
| FGFR4 | BCAM |
| FGR | BCAR1 |
| FLI1 | BCAR3 |
| FLT3 | BCAS1 |
| FOS | BCL10 |
| FOSB | BCL11A |
| FOXO1 | BCL11B |
| FOXO3 | BCL2 |
| FOXO4 | BCL2A1 |
| FOXP1 | BCL2L1 |
| FUS | BCL2L11 |
| GAS7 | BCL3 |
| GLI1 | BCL6 |
| GLIS2 | BCL7A |
| HDAC10 | BCL9 |
| HERPUD1 | BCOR |
| HEY1 | BCR |
| HIP1 | BIN2 |
| HLF | BIRC3 |
| HMGA1 | BIRC5 |
| HMGA2 | BLK |
| IGH | BLM |
| IGK | BLNK |
| IGLL5 | BLVRA |
| IKZF1 | BMF |
| IKZF2 | BMP2 |
| IKZF3 | BMP4 |
| INSR | BMPR1A |
| IRF4 | BMPR1B |
| ITK | BNC2 |
| JAK1 | BRAF |
| JAK2 | BRCA1 |
| JAK3 | BRCA2 |
| JAZF1 | BRD3 |
| KAT6A | BRD4 |
| KIF5B | BRDT |
| KIT | BRINP3 |
| KMT2A | BRIP1 |
| LATS2 | BRPF1 |
| LCP1 | BTG1 |
| LMNA | BTG3 |
| LMO1 | BTK |
| LMO2 | BTLA |
| LPP | BUB1 |
| LTK | BUB1B |
| MAF | C15orf48 |
| MAFB | C1RL |
| MALT1 | C3 |
| MAML2 | C5 |
| MAP3K7 | C5AR2 |
| MAP3K8 | CA4 |
| MAST1 | CAGE1 |
| MDS2 | CALB2 |
| MECOM | CALHM6 |
| MEF2C | CALML3 |
| MEF2D | CALR |
| MEN1 | CAMTA1 |
| MET | CANX |
| MGMT | CASP1 |
| MKL1 | CASP3 |
| MKL2 | CASP8 |
| MLF1 | CASP9 |
| MLH1 | CAVIN1 |
| MLLT1 | CBFA2T3 |
| MLLT10 | CBFB |
| MN1 | CBL |
| MNX1 | CBLC |
| MSH2 | CCDC140 |
| MSH6 | CCDC50 |
| MSI2 | CCL11 |
| MSMB | CCL13 |
| MTAP | CCL14 |
| MTCP1 | CCL17 |
| MYB | CCL18 |
| MYC | CCL19 |
| MYH11 | CCL2 |
| NAB2 | CCL20 |
| NBEAP1 | CCL21 |
| NCOA2 | CCL3 |
| NF1 | CCL4 |
| NF2 | CCL5 |
| NIN | CCL8 |
| NONO | CCN2 |
| NOTCH1 | CCNA2 |
| NOTCH2 | CCNB1 |
| NOTCH3 | CCNB2 |
| NOTCH4 | CCND1 |
| NPM1 | CCND2 |
| NR4A3 | CCND3 |
| NRAS | CCNE1 |
| NRG1 | CCNE2 |
| NRG2 | CCNG2 |
| NSD1 | CCR4 |
| NTRK1 | CCR5 |
| NTRK2 | CCR7 |
| NTRK3 | CCR8 |
| NUMA1 | CCRL2 |
| NUP214 | CCSER2 |
| NUP98 | CD14 |
| NUTM1 | CD163 |
| NUTM2A | CD19 |
| P2RY8 | CD1A |
| PALB2 | CD1B |
| PAX3 | CD1D |
| PAX5 | CD1E |
| PAX7 | CD2 |
| PBX1 | CD209 |
| PBX3 | CD22 |
| PDCD1LG2 | CD226 |
| PDGFB | CD244 |
| PDGFD | CD247 |
| PDGFRA | CD248 |
| PDGFRB | CD27 |
| PHF1 | CD274 |
| PICALM | CD276 |
| PIK3CA | CD28 |
| PIK3R1 | CD33 |
| PIK3R2 | CD34 |
| PKN1 | CD36 |
| PLAG1 | CD38 |
| PMS2 | CD3D |
| PPARG | CD3E |
| PRDM16 | CD3G |
| PRKACA | CD4 |
| PRKCA | CD40 |
| PRKCB | CD40LG |
| PRMT5 | CD44 |
| PTCH1 | CD46 |
| PTEN | CD47 |
| RAF1 | CD5 |
| RARA | CD6 |
| RASGRF1 | CD63 |
| RB1 | CD68 |
| RELA | CD7 |
| RET | CD70 |
| RHOH | CD74 |
| ROS1 | CD79A |
| RPS6KB1 | CD79B |
| RSPO2 | CD80 |
| RSPO3 | CD81 |
| RUNX1 | CD84 |
| RUNX1T1 | CD86 |
| SDHA | CD8A |
| SDHB | CD8B |
| SDHC | CD9 |
| SDHD | CD93 |
| SEC31A | CD96 |
| SEPT5 | CDC20 |
| SEPT6 | CDC25C |
| SEPT9 | CDC45 |
| SET | CDC6 |
| SH3GL1 | CDCA3 |
| SLC45A3 | CDCA5 |
| SMAD2 | CDCA7 |
| SMAD4 | CDCA7L |
| SMARCB1 | CDCA8 |
| SNX29 | CDH1 |
| SRC | CDH3 |
| SS18 | CDH5 |
| SSX1 | CDHR1 |
| SSX2 | CDK2 |
| SSX4 | CDK4 |
| STAT6 | CDK6 |
| STIL | CDK8 |
| STK11 | CDKN1A |
| SYK | CDKN1B |
| TAF15 | CDKN1C |
| TAL1 | CDKN2A |
| TAL2 | CDKN2AIP |
| TBL1XR1 | CDKN2B |
| TCF3 | CDKN2B-AS1 |
| TCF4 | CDKN2D |
| TCL1A | CDKN3 |
| TCL6 | CDT1 |
| TEC | CDX2 |
| TERT | CEACAM1 |
| TET1 | CEACAM3 |
| TET2 | CEACAM5 |
| TFE3 | CEACAM8 |
| TFEB | CEBPA |
| TFG | CEBPB |
| TFPT | CELSR2 |
| THADA | CENPA |
| TLX1 | CENPF |
| TLX3 | CENPM |
| TMPRSS2 | CEP43 |
| TOP1 | CEP55 |
| TP53 | CES1 |
| TP63 | CES2 |
| TPM3 | CFD |
| TPM4 | CGAS |
| TRIM24 | CHAF1B |
| TRIP11 | CHEK1 |
| TSC1 | CHEK2 |
| TSC2 | CHN1 |
| TTL | CHUK |
| TYK2 | CIC |
| USP6 | CIITA |
| VHL | CITED4 |
| WHSC1 | CLCA2 |
| WHSC1L1 | CLDN18 |
| WT1 | CLDN3 |
| WWTR1 | CLDN4 |
| YAP1 | CLDN5 |
| YWHAE | CLDN6 |
| ZAP70 | CLDN7 |
| ZBTB16 | CLEC10A |
| ZMYM2 | CLEC14A |
| ZNF384 | CLEC4C |
| ZNF750 | CLEC5A |
|  | CLEC9A |
|  | CLIC2 |
|  | CLIC4 |
|  | CLTC |
|  | CMC4 |
|  | CMKLR1 |
|  | CMPK2 |
|  | CNN1 |
|  | CNTNAP2 |
|  | CNTRL |
|  | COL15A1 |
|  | COL18A1 |
|  | COL1A1 |
|  | COL1A2 |
|  | COL3A1 |
|  | COL4A1 |
|  | COL4A2 |
|  | COL6A3 |
|  | COL7A1 |
|  | COPB2 |
|  | CPA3 |
|  | CRAT |
|  | CREB1 |
|  | CREB3L1 |
|  | CREB3L2 |
|  | CREBBP |
|  | CRKL |
|  | CRLF2 |
|  | CRNDE |
|  | CRYAB |
|  | CSF1 |
|  | CSF1R |
|  | CSF2 |
|  | CSF3R |
|  | CSMD1 |
|  | CSNK1E |
|  | CSNK1G2 |
|  | CST7 |
|  | CT45A1 |
|  | CT45A2 |
|  | CT45A3 |
|  | CT62 |
|  | CTAG1A |
|  | CTAG1B |
|  | CTAG2 |
|  | CTAGE1 |
|  | CTLA4 |
|  | CTNNB1 |
|  | CTNNBIP1 |
|  | CTPS1 |
|  | CTPS2 |
|  | CTSV |
|  | CTSW |
|  | CUX1 |
|  | CX3CL1 |
|  | CXCL1 |
|  | CXCL10 |
|  | CXCL11 |
|  | CXCL12 |
|  | CXCL13 |
|  | CXCL2 |
|  | CXCL3 |
|  | CXCL6 |
|  | CXCL8 |
|  | CXCL9 |
|  | CXCR1 |
|  | CXCR2 |
|  | CXCR4 |
|  | CXCR5 |
|  | CXCR6 |
|  | CXXC5 |
|  | CYB5R2 |
|  | CYBB |
|  | CYLD |
|  | CYP4F3 |
|  | DCAF12 |
|  | DCLK1 |
|  | DCN |
|  | DDB2 |
|  | DDIT3 |
|  | DDIT4 |
|  | DDR1 |
|  | DDR2 |
|  | DDX10 |
|  | DDX21 |
|  | DDX4 |
|  | DDX58 |
|  | DDX6 |
|  | DEK |
|  | DENND3 |
|  | DEPTOR |
|  | DHH |
|  | DHX58 |
|  | DIDO1 |
|  | DKK1 |
|  | DKK2 |
|  | DKK4 |
|  | DLC1 |
|  | DLL3 |
|  | DLL4 |
|  | DMBT1 |
|  | DMD |
|  | DNMT1 |
|  | DNMT3A |
|  | DOCK5 |
|  | DOT1L |
|  | DRAM1 |
|  | DSC2 |
|  | DSCR8 |
|  | DTL |
|  | DTX1 |
|  | DTX2 |
|  | DTX3L |
|  | DUSP1 |
|  | DUSP18 |
|  | DUSP22 |
|  | DUSP6 |
|  | DVL1 |
|  | E2F1 |
|  | E2F4 |
|  | E2F5 |
|  | EBF1 |
|  | ECSCR |
|  | ECT2 |
|  | EDNRB |
|  | EDNRB-AS1 |
|  | EGF |
|  | EGFR |
|  | EGLN3 |
|  | EGR1 |
|  | EGR2 |
|  | EIF4A2 |
|  | ELF4 |
|  | ELF5 |
|  | ELK4 |
|  | ELL |
|  | ELN |
|  | EMCN |
|  | EME1 |
|  | EML4 |
|  | EML6 |
|  | EMSY |
|  | ENTPD1 |
|  | EOMES |
|  | EP300 |
|  | EP400 |
|  | EPCAM |
|  | EPHA4 |
|  | EPHA7 |
|  | EPOR |
|  | EPS15 |
|  | ERAP1 |
|  | ERAP2 |
|  | ERBB2 |
|  | ERBB3 |
|  | ERCC1 |
|  | ERCC2 |
|  | ERCC3 |
|  | ERCC4 |
|  | ERCC5 |
|  | EREG |
|  | ERG |
|  | ERN2 |
|  | ESM1 |
|  | ESR1 |
|  | ETS1 |
|  | ETV1 |
|  | ETV4 |
|  | ETV5 |
|  | ETV6 |
|  | EWSR1 |
|  | EXO1 |
|  | EZH2 |
|  | F11R |
|  | FAM171B |
|  | FAM241B |
|  | FAM30A |
|  | FANCA |
|  | FANCB |
|  | FANCC |
|  | FANCD2 |
|  | FAP |
|  | FAS |
|  | FASN |
|  | FAT2 |
|  | FBXW11 |
|  | FBXW7 |
|  | FCAR |
|  | FCGR2B |
|  | FCGR3B |
|  | FCRL2 |
|  | FCRL5 |
|  | FEV |
|  | FGF9 |
|  | FGFBP2 |
|  | FGFR1 |
|  | FGFR2 |
|  | FGFR3 |
|  | FGFR4 |
|  | FGR |
|  | FKBP4 |
|  | FLI1 |
|  | FLNA |
|  | FLT1 |
|  | FLT3 |
|  | FLT3LG |
|  | FLT4 |
|  | FMN1 |
|  | FMN2 |
|  | FMOD |
|  | FN1 |
|  | FNBP1 |
|  | FNIP2 |
|  | FOLH1 |
|  | FOLR1 |
|  | FOS |
|  | FOSB |
|  | FOXA1 |
|  | FOXC1 |
|  | FOXM1 |
|  | FOXO1 |
|  | FOXO3 |
|  | FOXO4 |
|  | FOXO6 |
|  | FOXP1 |
|  | FOXP3 |
|  | FPR1 |
|  | FPR3 |
|  | FSTL3 |
|  | FUCA1 |
|  | FUS |
|  | FUT4 |
|  | FUT8 |
|  | FZD1 |
|  | FZD10 |
|  | FZD2 |
|  | FZD5 |
|  | FZD6 |
|  | FZD7 |
|  | GABBR2 |
|  | GADD45A |
|  | GADD45B |
|  | GAGE1 |
|  | GAGE2E |
|  | GAGE6 |
|  | GAGE8 |
|  | GALNT10 |
|  | GALNT12 |
|  | GALNT17 |
|  | GAS1 |
|  | GAS7 |
|  | GBP5 |
|  | GIMAP5 |
|  | GIMAP7 |
|  | GINS2 |
|  | GJA4 |
|  | GLI1 |
|  | GLIS2 |
|  | GMFG |
|  | GMNN |
|  | GMPS |
|  | GNA12 |
|  | GNG11 |
|  | GNLY |
|  | GOLM1 |
|  | GPA33 |
|  | GPC4 |
|  | GPC6 |
|  | GPI |
|  | GPR143 |
|  | GPR146 |
|  | GPR160 |
|  | GRB7 |
|  | GREB1 |
|  | GRM4 |
|  | GSK3B |
|  | GSTA1 |
|  | GSTM1 |
|  | GUSB |
|  | GZMA |
|  | GZMB |
|  | GZMH |
|  | GZMK |
|  | H1-1 |
|  | H1-4 |
|  | H2AC11 |
|  | H2AC13 |
|  | H2AX |
|  | H2BC13 |
|  | H2BC18 |
|  | H3C2 |
|  | HABP2 |
|  | HAMP |
|  | HAP1 |
|  | HAVCR2 |
|  | HBEGF |
|  | HCLS1 |
|  | HCST |
|  | HDAC1 |
|  | HDAC10 |
|  | HDAC11 |
|  | HDAC2 |
|  | HDAC3 |
|  | HDAC4 |
|  | HDAC5 |
|  | HDAC6 |
|  | HDAC7 |
|  | HDAC8 |
|  | HDAC9 |
|  | HDC |
|  | HELZ2 |
|  | HERPUD1 |
|  | HES1 |
|  | HES2 |
|  | HES4 |
|  | HES5 |
|  | HES6 |
|  | HEY1 |
|  | HEY2 |
|  | HEYL |
|  | HGF |
|  | HHIP |
|  | HIF1A |
|  | HIP1 |
|  | HLA-A |
|  | HLA-B |
|  | HLA-C |
|  | HLA-DMA |
|  | HLA-DMB |
|  | HLA-DOA |
|  | HLA-DOB |
|  | HLA-DQA1 |
|  | HLA-DQB1 |
|  | HLA-DRA |
|  | HLA-DRB1 |
|  | HLA-E |
|  | HLF |
|  | HMGA1 |
|  | HMGA2 |
|  | HMGCS2 |
|  | HMMR |
|  | HOPX |
|  | HORMAD1 |
|  | HOXA11 |
|  | HOXB2 |
|  | HPCAL1 |
|  | HRAS |
|  | HSD11B1 |
|  | HSP90AA1 |
|  | HSP90AB1 |
|  | HSPA4L |
|  | HSPB1 |
|  | ICAM1 |
|  | ICAM2 |
|  | ICOS |
|  | ID1 |
|  | ID2 |
|  | IDO1 |
|  | IFI16 |
|  | IFI27 |
|  | IFI35 |
|  | IFI6 |
|  | IFIT1 |
|  | IFIT2 |
|  | IFIT3 |
|  | IFITM2 |
|  | IFITM3 |
|  | IFNG |
|  | IFNL2 |
|  | IGF1 |
|  | IGF1R |
|  | IGFBP1 |
|  | IGFBP3 |
|  | IGFBP4 |
|  | IGLL5 |
|  | IHH |
|  | IKBKE |
|  | IKZF1 |
|  | IKZF2 |
|  | IKZF3 |
|  | IL10 |
|  | IL11 |
|  | IL12A |
|  | IL13 |
|  | IL13RA2 |
|  | IL15 |
|  | IL16 |
|  | IL17RA |
|  | IL1A |
|  | IL1B |
|  | IL1R1 |
|  | IL1RN |
|  | IL21R |
|  | IL23A |
|  | IL2RA |
|  | IL3 |
|  | IL33 |
|  | IL3RA |
|  | IL4R |
|  | IL6 |
|  | IL6R |
|  | IL6ST |
|  | IL7 |
|  | IL7R |
|  | IMPDH1 |
|  | INPP1 |
|  | INSR |
|  | INSRR |
|  | IPO8 |
|  | IQGAP3 |
|  | IRAG1 |
|  | IRAG2 |
|  | IRF1 |
|  | IRF4 |
|  | IRF7 |
|  | IRF8 |
|  | IRGM |
|  | IRS2 |
|  | IRX4 |
|  | ISG20 |
|  | ISY1 |
|  | ITFG2 |
|  | ITGAM |
|  | ITGAV |
|  | ITGAX |
|  | ITGB1 |
|  | ITGB2 |
|  | ITGB4 |
|  | ITK |
|  | ITM2A |
|  | ITPKB |
|  | JAK1 |
|  | JAK2 |
|  | JAK3 |
|  | JAML |
|  | JAZF1 |
|  | JCAD |
|  | JUN |
|  | KAT6A |
|  | KCNE3 |
|  | KCNJ15 |
|  | KCNK5 |
|  | KCNMA1 |
|  | KDM1A |
|  | KDM3B |
|  | KDM4C |
|  | KDM5C |
|  | KDM5D |
|  | KDR |
|  | KDSR |
|  | KIAA0040 |
|  | KIAA0319L |
|  | KIF13B |
|  | KIF23 |
|  | KIF2B |
|  | KIF2C |
|  | KIF5B |
|  | KIFC1 |
|  | KIR2DL1 |
|  | KIR2DL3 |
|  | KIR3DL1 |
|  | KIR3DL2 |
|  | KIR3DS1 |
|  | KIT |
|  | KLF2 |
|  | KLF4 |
|  | KLK3 |
|  | KLRB1 |
|  | KLRC3 |
|  | KLRC4 |
|  | KLRD1 |
|  | KLRK1 |
|  | KMT2A |
|  | KMT2C |
|  | KMT2D |
|  | KMT5A |
|  | KRAS |
|  | KRT14 |
|  | KRT17 |
|  | KRT31 |
|  | KRT5 |
|  | KRT6A |
|  | KRTCAP3 |
|  | KYNU |
|  | LAG3 |
|  | LAIR1 |
|  | LAMB1 |
|  | LASP1 |
|  | LATS1 |
|  | LATS2 |
|  | LCK |
|  | LCN2 |
|  | LCP1 |
|  | LDHB |
|  | LEF1 |
|  | LGALS2 |
|  | LGALS3 |
|  | LILRB5 |
|  | LIMD1 |
|  | LIMK2 |
|  | LINC-ROR |
|  | LINC00598 |
|  | LIPH |
|  | LIPI |
|  | LMNA |
|  | LMO1 |
|  | LMO2 |
|  | LMO3 |
|  | LMO4 |
|  | LOC100506207 |
|  | LOC100507346 |
|  | LPP |
|  | LRP1 |
|  | LRP8 |
|  | LRRC15 |
|  | LTF |
|  | LTK |
|  | LUZP4 |
|  | LY6E |
|  | LY6G6D |
|  | LYL1 |
|  | LZTR1 |
|  | MACC1 |
|  | MAF |
|  | MAFB |
|  | MAGEA1 |
|  | MAGEA10 |
|  | MAGEA11 |
|  | MAGEA12 |
|  | MAGEA2B |
|  | MAGEA3 |
|  | MAGEA4 |
|  | MAGEA5P |
|  | MAGEA6 |
|  | MAGEA8 |
|  | MAGEA9B |
|  | MAGEB1 |
|  | MAGEB10 |
|  | MAGEB16 |
|  | MAGEB17 |
|  | MAGEB18 |
|  | MAGEB2 |
|  | MAGEB3 |
|  | MAGEB4 |
|  | MAGEB5 |
|  | MAGEB6 |
|  | MAGEC1 |
|  | MAGEC2 |
|  | MAGEC3 |
|  | MALAT1 |
|  | MALT1 |
|  | MAML2 |
|  | MAML3 |
|  | MAP2 |
|  | MAP2K1 |
|  | MAP2K3 |
|  | MAP3K21 |
|  | MAP3K7 |
|  | MAP3K8 |
|  | MAP4K4 |
|  | MAPK1 |
|  | MAPK3 |
|  | MAPKAPK2 |
|  | MAPT |
|  | MARK1 |
|  | MASP2 |
|  | MAST1 |
|  | MAST2 |
|  | MASTL |
|  | MBTD1 |
|  | MCAM |
|  | MCL1 |
|  | MCM10 |
|  | MCM2 |
|  | MCM4 |
|  | MCM6 |
|  | MDC1 |
|  | MDM2 |
|  | MDS2 |
|  | MECOM |
|  | MEF2C |
|  | MEF2D |
|  | MEG3 |
|  | MEGF9 |
|  | MELK |
|  | MEN1 |
|  | MEST |
|  | MET |
|  | METRNL |
|  | MFAP4 |
|  | MFAP5 |
|  | MGA |
|  | MGMT |
|  | MGST2 |
|  | MIA |
|  | MIAT |
|  | MICB |
|  | MIR100 |
|  | MITF |
|  | MKI67 |
|  | MLF1 |
|  | MLH1 |
|  | MLLT1 |
|  | MLLT10 |
|  | MLPH |
|  | MME |
|  | MMP11 |
|  | MN1 |
|  | MNX1 |
|  | MOCOS |
|  | MPZL3 |
|  | MRAS |
|  | MRE11 |
|  | MRTFA |
|  | MRTFB |
|  | MS4A1 |
|  | MS4A2 |
|  | MS4A4A |
|  | MSH2 |
|  | MSH6 |
|  | MSI2 |
|  | MSMB |
|  | MSN |
|  | MST1R |
|  | MTAP |
|  | MTHFD1L |
|  | MTOR |
|  | MUC1 |
|  | MUC16 |
|  | MUTYH |
|  | MVP |
|  | MX1 |
|  | MX2 |
|  | MYB |
|  | MYBL2 |
|  | MYC |
|  | MYCL |
|  | MYCN |
|  | MYCT1 |
|  | MYD88 |
|  | MYH11 |
|  | MYH9 |
|  | NAB2 |
|  | NAT1 |
|  | NAV3 |
|  | NBEA |
|  | NBEAP1 |
|  | NBN |
|  | NCAM1 |
|  | NCOA2 |
|  | NCOR1 |
|  | NCR1 |
|  | NDC80 |
|  | NDE1 |
|  | NDRG1 |
|  | NEAT1 |
|  | NECTIN1 |
|  | NECTIN2 |
|  | NECTIN3 |
|  | NEK1 |
|  | NEK2 |
|  | NEK6 |
|  | NELL2 |
|  | NF1 |
|  | NF2 |
|  | NFATC2 |
|  | NFE2L2 |
|  | NFIC |
|  | NFKB2 |
|  | NID2 |
|  | NIN |
|  | NKD1 |
|  | NKG7 |
|  | NKX3-1 |
|  | NLK |
|  | NONO |
|  | NOS1 |
|  | NOS1AP |
|  | NOTCH1 |
|  | NOTCH2 |
|  | NOTCH3 |
|  | NOTCH4 |
|  | NPAS2 |
|  | NPM1 |
|  | NR4A3 |
|  | NRAP |
|  | NRARP |
|  | NRAS |
|  | NRG1 |
|  | NRG2 |
|  | NRP1 |
|  | NRP2 |
|  | NRTN |
|  | NSD1 |
|  | NSD2 |
|  | NSD3 |
|  | NT5C3A |
|  | NT5E |
|  | NTRK1 |
|  | NTRK2 |
|  | NTRK3 |
|  | NUF2 |
|  | NUMA1 |
|  | NUMBL |
|  | NUP214 |
|  | NUP98 |
|  | NUTM1 |
|  | NUTM2A |
|  | NXF2B |
|  | NXPH3 |
|  | OAS3 |
|  | OASL |
|  | ODC1 |
|  | OGN |
|  | OLFM1 |
|  | OLFM4 |
|  | OLIG2 |
|  | ORAI2 |
|  | ORC6 |
|  | P2RY8 |
|  | PADI2 |
|  | PAFAH1B2 |
|  | PAGE5 |
|  | PAK2 |
|  | PAK4 |
|  | PALB2 |
|  | PAMR1 |
|  | PARP1 |
|  | PARP12 |
|  | PARP14 |
|  | PAX3 |
|  | PAX5 |
|  | PAX7 |
|  | PAX8 |
|  | PBK |
|  | PBX1 |
|  | PBX3 |
|  | PCDH17 |
|  | PCSK1 |
|  | PDCD1 |
|  | PDCD1LG2 |
|  | PDGFA |
|  | PDGFB |
|  | PDGFD |
|  | PDGFRA |
|  | PDGFRB |
|  | PDIA3 |
|  | PDZK1IP1 |
|  | PECAM1 |
|  | PFN2 |
|  | PGR |
|  | PHF1 |
|  | PHF11 |
|  | PHGDH |
|  | PHLPP1 |
|  | PICALM |
|  | PIK3CA |
|  | PIK3CD |
|  | PIK3CG |
|  | PIK3R1 |
|  | PIK3R2 |
|  | PIM2 |
|  | PIM3 |
|  | PIMREG |
|  | PIP4P1 |
|  | PKN1 |
|  | PLA2G7 |
|  | PLAAT1 |
|  | PLAC8 |
|  | PLAG1 |
|  | PLAGL2 |
|  | PLCB4 |
|  | PLEK2 |
|  | PLEKHA4 |
|  | PLEKHB1 |
|  | PLK2 |
|  | PLPP3 |
|  | PLVAP |
|  | PMEPA1 |
|  | PML |
|  | PMS1 |
|  | PMS2 |
|  | PNOC |
|  | PNPLA7 |
|  | PODXL |
|  | POLD1 |
|  | POLE |
|  | POU2F2 |
|  | POU5F1 |
|  | PPARG |
|  | PPM1J |
|  | PPP1R13L |
|  | PRDM15 |
|  | PRDM16 |
|  | PRF1 |
|  | PRKACA |
|  | PRKACB |
|  | PRKACG |
|  | PRKCA |
|  | PRKCB |
|  | PRMT1 |
|  | PRMT5 |
|  | PRND |
|  | PROM1 |
|  | PRPF6 |
|  | PRPF8 |
|  | PRR29 |
|  | PSAT1 |
|  | PSCA |
|  | PSD3 |
|  | PSENEN |
|  | PSIP1 |
|  | PSMB10 |
|  | PSMB8 |
|  | PSMB9 |
|  | PSME1 |
|  | PTCH1 |
|  | PTCH2 |
|  | PTCRA |
|  | PTEN |
|  | PTGDS |
|  | PTGER2 |
|  | PTGER4 |
|  | PTGS2 |
|  | PTPN1 |
|  | PTPN11 |
|  | PTPN22 |
|  | PTPRB |
|  | PTPRC |
|  | PTPRK |
|  | PTPRO |
|  | PTPRZ1 |
|  | PTTG1 |
|  | PUM1 |
|  | PVR |
|  | PVRIG |
|  | PXDC1 |
|  | R3HDM1 |
|  | RAB23 |
|  | RAB27A |
|  | RAB29 |
|  | RAC1 |
|  | RAD50 |
|  | RAD51 |
|  | RAD51AP1 |
|  | RAD51B |
|  | RAD51C |
|  | RAD51D |
|  | RAD52 |
|  | RAD54L |
|  | RAF1 |
|  | RAPGEFL1 |
|  | RARA |
|  | RASGRF1 |
|  | RASIP1 |
|  | RASSF6 |
|  | RB1 |
|  | RBL1 |
|  | RBM24 |
|  | RBP7 |
|  | RBX1 |
|  | RECQL4 |
|  | REG4 |
|  | RELA |
|  | RERG |
|  | RET |
|  | RFLNB |
|  | RGCC |
|  | RGS10 |
|  | RGS16 |
|  | RGS2 |
|  | RHOA |
|  | RHOH |
|  | RHOJ |
|  | RIT1 |
|  | RNF13 |
|  | ROBO4 |
|  | ROCK2 |
|  | ROPN1 |
|  | ROPN1B |
|  | ROR1 |
|  | RORA |
|  | RORC |
|  | ROS1 |
|  | RP1 |
|  | RPL23 |
|  | RPL39L |
|  | RPS26 |
|  | RPS6KA1 |
|  | RPS6KB1 |
|  | RPSAP52 |
|  | RRAGC |
|  | RRAS |
|  | RRM2 |
|  | RSAD2 |
|  | RSPO2 |
|  | RSPO3 |
|  | RUNX1 |
|  | RUNX1T1 |
|  | RUNX2 |
|  | RUNX3 |
|  | S100A12 |
|  | S100A8 |
|  | S1PR2 |
|  | SAA1 |
|  | SAGE1 |
|  | SAMD9L |
|  | SAP30 |
|  | SCD |
|  | SCD5 |
|  | SCML4 |
|  | SCUBE2 |
|  | SDC1 |
|  | SDHA |
|  | SDHB |
|  | SDHC |
|  | SDHD |
|  | SEC31A |
|  | SELENOW |
|  | SELL |
|  | SELP |
|  | SEMA3E |
|  | SEMA4B |
|  | SEMA4C |
|  | SEMA6D |
|  | SEMA7A |
|  | SEPTIN12 |
|  | SEPTIN5 |
|  | SEPTIN6 |
|  | SEPTIN9 |
|  | SERPINA9 |
|  | SERPINB13 |
|  | SERPINB2 |
|  | SERPINB5 |
|  | SERPINE1 |
|  | SERPINF1 |
|  | SESN1 |
|  | SESN2 |
|  | SESN3 |
|  | SET |
|  | SF3B1 |
|  | SFRP1 |
|  | SGK3 |
|  | SH2D1A |
|  | SH2D1B |
|  | SH2D2A |
|  | SH3BP5 |
|  | SH3GL1 |
|  | SH3PXD2A |
|  | SHCBP1 |
|  | SHISA5 |
|  | SHISA8 |
|  | SHOC2 |
|  | SIGLEC5 |
|  | SKP1 |
|  | SLAMF1 |
|  | SLC16A3 |
|  | SLC1A2 |
|  | SLC22A8 |
|  | SLC39A6 |
|  | SLC40A1 |
|  | SLC45A3 |
|  | SLC49A4 |
|  | SLC7A8 |
|  | SLC9A3R1 |
|  | SLCO2A1 |
|  | SLFN11 |
|  | SLIT2 |
|  | SMAD2 |
|  | SMAD3 |
|  | SMAD4 |
|  | SMAD9 |
|  | SMARCB1 |
|  | SMURF2 |
|  | SNAI1 |
|  | SNRNP70 |
|  | SNW1 |
|  | SNX29 |
|  | SOCS1 |
|  | SOS1 |
|  | SOS2 |
|  | SOX11 |
|  | SOX17 |
|  | SOX18 |
|  | SOX9 |
|  | SP2-AS1 |
|  | SPANXA1 |
|  | SPANXB1 |
|  | SPANXC |
|  | SPARC |
|  | SPARCL1 |
|  | SPIB |
|  | SPINK1 |
|  | SPN |
|  | SPP1 |
|  | SPRY4 |
|  | SRC |
|  | SRD5A1 |
|  | SREBF1 |
|  | SRSF3 |
|  | SS18 |
|  | SSPOP |
|  | SSX1 |
|  | SSX2 |
|  | SSX2B |
|  | SSX3 |
|  | SSX4 |
|  | SSX5 |
|  | ST3GAL2 |
|  | STAT1 |
|  | STAT3 |
|  | STAT4 |
|  | STAT6 |
|  | STAU2 |
|  | STEAP1 |
|  | STEAP4 |
|  | STIL |
|  | STING1 |
|  | STK11 |
|  | STON1 |
|  | SULF2 |
|  | SULT1A1 |
|  | SUV39H2 |
|  | SYCP1 |
|  | SYCP3 |
|  | SYK |
|  | TACSTD2 |
|  | TAF15 |
|  | TAGAP |
|  | TAGLN |
|  | TAL1 |
|  | TAL2 |
|  | TAP1 |
|  | TAP2 |
|  | TAPBP |
|  | TBC1D10C |
|  | TBC1D4 |
|  | TBC1D9 |
|  | TBL1XR1 |
|  | TBX21 |
|  | TCF12 |
|  | TCF3 |
|  | TCF4 |
|  | TCF7L1 |
|  | TCF7L2 |
|  | TCL1A |
|  | TCL6 |
|  | TDG |
|  | TDGF1 |
|  | TDRD7 |
|  | TEAD1 |
|  | TEC |
|  | TEK |
|  | TENM3 |
|  | TENT5A |
|  | TERC |
|  | TERT |
|  | TET1 |
|  | TET2 |
|  | TET3 |
|  | TFCP2L1 |
|  | TFE3 |
|  | TFEB |
|  | TFF1 |
|  | TFG |
|  | TFPT |
|  | TFRC |
|  | TGFB1 |
|  | TGFB2 |
|  | TGFB3 |
|  | TGFBI |
|  | TGFBR1 |
|  | TGFBR2 |
|  | THADA |
|  | THBD |
|  | THBS1 |
|  | THY1 |
|  | TIAM1 |
|  | TIE1 |
|  | TIGIT |
|  | TIMP3 |
|  | TLL1 |
|  | TLR2 |
|  | TLR3 |
|  | TLX1 |
|  | TLX3 |
|  | TMEM38A |
|  | TMEM45B |
|  | TMPRSS2 |
|  | TNF |
|  | TNFRSF10C |
|  | TNFRSF11A |
|  | TNFRSF14 |
|  | TNFRSF17 |
|  | TNFRSF1A |
|  | TNFRSF1B |
|  | TNFRSF25 |
|  | TNFRSF8 |
|  | TNFRSF9 |
|  | TNFSF10 |
|  | TNFSF11 |
|  | TNFSF12 |
|  | TNFSF13B |
|  | TNFSF4 |
|  | TNFSF9 |
|  | TNKS |
|  | TNKS2 |
|  | TNS1 |
|  | TOP1 |
|  | TOP2A |
|  | TP53 |
|  | TP53BP1 |
|  | TP53INP1 |
|  | TP53INP2 |
|  | TP63 |
|  | TP73 |
|  | TPM1 |
|  | TPM2 |
|  | TPM3 |
|  | TPM4 |
|  | TPSAB1 |
|  | TPSB2 |
|  | TPST1 |
|  | TPX2 |
|  | TRAT1 |
|  | TREM2 |
|  | TREX1 |
|  | TRG-AS1 |
|  | TRIM2 |
|  | TRIM24 |
|  | TRIM56 |
|  | TRIP11 |
|  | TRPS1 |
|  | TSC1 |
|  | TSC2 |
|  | TSHR |
|  | TTC39B |
|  | TTK |
|  | TTL |
|  | TTTY14 |
|  | TTYH1 |
|  | TWIST1 |
|  | TYK2 |
|  | TYMS |
|  | UBA7 |
|  | UBE2C |
|  | UBE2T |
|  | UBXN4 |
|  | UGT8 |
|  | UNC5B |
|  | UPK1A |
|  | UPP1 |
|  | USP44 |
|  | USP6 |
|  | USP8 |
|  | VAV3 |
|  | VCAM1 |
|  | VCL |
|  | VEGFA |
|  | VEGFB |
|  | VEGFC |
|  | VGLL1 |
|  | VHL |
|  | VIM |
|  | VNN3P |
|  | VPREB1 |
|  | VWF |
|  | WASH5P |
|  | WIF1 |
|  | WNT11 |
|  | WNT16 |
|  | WNT2 |
|  | WNT5B |
|  | WNT7A |
|  | WNT7B |
|  | WNT8B |
|  | WT1 |
|  | WWTR1 |
|  | XCL1 |
|  | XCL2 |
|  | XIST |
|  | XPA |
|  | XPO1 |
|  | YAP1 |
|  | YWHAE |
|  | YY1 |
|  | ZAP70 |
|  | ZBP1 |
|  | ZBTB16 |
|  | ZBTB46 |
|  | ZC3H13 |
|  | ZC3HAV1 |
|  | ZEB1 |
|  | ZEB2 |
|  | ZIC2 |
|  | ZMAT3 |
|  | ZMYM2 |
|  | ZNF384 |
|  | ZNF521 |
|  | ZNF608 |
|  | ZNF703 |
|  | ZNF750 |
|  | ZNRF3 |

**Table S2. List of genes with actionable fusion evaluated in accuracy study**

| **Genes with Actionable Fusion** | **Actionable Fusions** | | | |
| --- | --- | --- | --- | --- |
| *ABL1* | *BCR-ABL1* |  |  |  |
| *ALK* | *ATIC-ALK* | *EML4-ALK* | *KANK1-ALK* | *NPM1-ALK* |
| *BRAF* | *KIAA1549-BRAF* | *MKRN1-BRAF* | *MYCBP2-BRAF* |  |
| *FGFR1* | *FGFR1-TACC1* |  |  |  |
| *FGFR2* | *FGFR2-CCDC6* | *FGFR2-IKZF2* | *UGGT1-FGFR2* |  |
| *FGFR3* | *FGFR3-TACC3* |  |  |  |
| *JAK2* | *PAX5-JAK2* |  |  |  |
| *NTRK1* | *LMNA-NTRK1* | *TPM3-NTRK1* | *TPR-NTRK1* |  |
| *NTRK3* | *EML4-NTRK3* |  |  |  |
| *PDGFRA* | *GP1-PDGFRA* |  |  |  |
| *PIK3CA* | *TBL1XR1-PIK3CA* |  |  |  |
| *RET* | *C10orf118-RET* | *KIF5B-RET* | *CCDC6-RET* |  |
| *ROS1* | *EZR-ROS1* | *CD74-ROS1* | *CEP290-ROS1* |  |

**Table S3. List of diagnostic fusions evaluated in accuracy study**

| **Diagnostic Fusions** | |
| --- | --- |
| *NPM1-ALK* | *KIAA1549-BRAF* |
| *CIC-DUX4* | *NAB2-STAT6* |
| *ESR1-NCOA2* | *NCOA2-TEAD1* |
| *EWSR1-ATF1* | *PAX3-FOXO1* |
| *EWSR1-ERG* | *SS18-SSX1* |
| *EWSR1-FLI1* | *TMPRSS2-ERG* |
| *EWSR1-KLF15* | *ASPSCR1-TFE3* |
| *EWSR1-TFCP2* | *NONO-TFE3* |
| *EWSR1-WT1* | *SFPQ-TFE3* |
| *FUS-DDIT3* | *PHF1-TFE3* |
| *FUS-TFCP2* | *HMGA2-MDM2* |
| *IGH-BCL2* | *HMGA2-NAV3* |
| *JAZF1-PHF1* | *HMGA2-HELB* |
| *JAZF1-SUZ12* | *HMGA2-SLC9A4* |

**Table S4. The concordance analysis result for all clinically relevant fusions stratified by sample type.**

| **Sample Type** | **Metric** | **PPA (%)** | **NPA (%)** | **PPV (%)** | **NPV (%)** | **OPA (%)** |
| --- | --- | --- | --- | --- | --- | --- |
| Clinical FFPE | Point Estimate | 88.57 | 99.99 | 96.88 | 99.95 | 99.93 |
|  | Count | (31/35) | (7416/7417) | (31/32) | (7416/7420) | (7447/7452) |
|  | 95% CI | [74.05, 95.46] | [99.92, 100.00] | [84.26, 99.45] | [99.86, 99.98] | [99.84, 99.97] |
| Clinical RNA Residual | Point Estimate | 89.25 | 99.79 | 84.69 | 99.86 | 99.66 |
|  | Count | (83/93) | (7160/7175) | (83/98) | (7160/7170) | (7243/7268) |
|  | 95% CI | [81.33, 94.05] | [99.66, 99.87] | [76.27, 90.50] | [99.74, 99.92] | [99.49, 99.77] |
| Total | Point Estimate | 89.06 | 99.89 | 87.69 | 99.90 | 99.80 |
|  | Count | (114/128) | (14576/14592) | (114/130) | (14576/14590) | (14690/14720) |
|  | 95% CI | [82.48, 93.37] | [99.82, 99.93] | [80.94, 92.28] | [99.84, 99.94] | [99.71, 99.86] |

PPA, NPA, PPV, NPV and OPA were estimated with 95% confidence interval (Wilson’s method)

**Table S5. The concordance analysis result for all clinically relevant fusions stratified by orthogonal assay type.**

| Orthogonal Assay | Metric | PPA (%) | NPA (%) | PPV (%) | NPV (%) | OPA (%) |
| --- | --- | --- | --- | --- | --- | --- |
| DNA-based Assay (F1, F1CDx or F1H DNA) | Point Estimate | 84.62 | 100.00 | 100.00 | 99.90 | 99.90 |
|  | Count | (22/26) | (3930/3930) | (22/22) | (3930/3934) | (3952/3956) |
|  | 95% CI | [66.47, 93.85] | [99.90, 100.00] | [85.13, 100.00] | [99.74, 99.96] | [99.74, 99.96] |
| RNA-based Assay (F1H RNA or Caris) | Point Estimate | 90.29 | 99.85 | 85.32 | 99.91 | 99.76 |
|  | Count | (93/103) | (10737/10753) | (93/109) | (10737/10747) | (10830/10856) |
|  | 95% CI | [83.04, 94.64] | [99.76, 99.91] | [77.48, 90.76] | [99.83,99.95] | [99.65, 99.84] |

PPA, NPA, PPV, NPV and OPA were estimated with 95% confidence interval (Wilson’s method). FoundationOne^®^ (F1), FoundationOne^®^CDx (F1CDx) and FoundationOne^®^Heme DNA (F1H DNA) are DNA-based NGS assays from Foundation Medicine. FoundationOne^®^Heme RNA (F1H RNA) is RNA-based NGS assay from Foundation Medicine. Caris is RNA-based NGS assay from Caris Life Sciences. Noted that, one FFPE sample (FM_RNASeq_033) was tested by both F1H DNA and F1H RNA workflow, this it was counted in both DNA-based and RNA-based assays in this analysis.

**Table S6. The concordance analysis result for all clinically relevant fusions stratified by tumor purity.**

| **Tumor Purity (%)** | **PPA (%)** | **NPA (%)** | **PPV (%)** | **NPV (%)** | **OPA (%)** |
| --- | --- | --- | --- | --- | --- |
| 20-29 | 85.71 (6/7) (48.69, 97.43) | 99.86 (728/729) (99.23, 99.98) | 85.71 (6/7) (48.69, 97.43) | 99.86 (728/729) (99.23, 99.98) | 99.73 (734/736) (99.01, 99.93) |
| 30-39 | 89.47 (17/19) (68.61, 97.06) | 99.86 (2921/2925) (99.65, 99.95) | 80.95 (17/21) (60, 92.33) | 99.93 (2921/2923) (99.75, 99.98) | 99.8 (2938/2944) (99.56, 99.91) |
| 40-49 | 87.5 (14/16) (63.98, 96.5) | 99.98 (4123/4124) (99.86, 100) | 93.33 (14/15) (70.18, 98.81) | 99.95 (4123/4125) (99.82, 99.99) | 99.93 (4137/4140) (99.79, 99.98) |
| 50-59 | 88.46 (23/26) (71.02, 96) | 99.91 (2180/2182) (99.67, 99.97) | 92 (23/25) (75.03, 97.78) | 99.86 (2180/2183) (99.6, 99.95) | 99.77 (2203/2208) (99.47, 99.9) |
| 60-69 | 90.91 (10/11) (62.26, 98.38) | 99.88 (816/817) (99.31, 99.98) | 90.91 (10/11) (62.26, 98.38) | 99.88 (816/817) (99.31, 99.98) | 99.76 (826/828) (99.12, 99.93) |
| 70-79 | 87.5 (21/24) (69, 95.66) | 99.95 (1907/1908) (99.7, 99.99) | 95.45 (21/22) (78.2, 99.19) | 99.84 (1907/1910) (99.54, 99.95) | 99.79 (1928/1932) (99.47, 99.92) |
| 80-89 | 100 (18/18) (82.41, 100) | 99.72 (1450/1454) (99.29, 99.89) | 81.82 (18/22) (61.48, 92.69) | 100 (1450/1450) (99.74, 100) | 99.73 (1468/1472) (99.3, 99.89) |
| 90-99 | 71.43 (5/7) (35.89, 91.78) | 99.56 (451/453) (98.4, 99.88) | 71.43 (5/7) (35.89, 91.78) | 99.56 (451/453) (98.4, 99.88) | 99.13 (456/460) (97.79, 99.66) |

PPA, NPA, PPV, NPV and OPA were estimated with 95% confidence interval (Wilson’s method) for each tumor purity bin.

**Table S7. The expanded result of LoD study.**

| **Cell Line** | **Fusion** | **Method** | **Minimum RNA Input** | **LoD** | **LoD Hit Rate** |
| --- | --- | --- | --- | --- | --- |
| K562 | BCR-ABL1* | Hit Rate | 1.5ng (0.5% input) | 33 | 100% (20/20) |
| LC-2/ad | CCDC6-RET* | Hit Rate | 15ng  (5% input) | 21 | 100% (20/20) |
| NCI-H2228 | EML4-ALK* | Hit Rate | 15ng (5% input) | 37 | 100% (20/20) |
| Reh | ETV6-RUNX1* | Hit Rate | 15ng  (5% input) | 37 | 100% (20/20) |
| NCI-H660 | TMPRSS2-ERG* | Hit Rate | 30ng  (10% input) | 21 | 100% (20/20) |
| NCI-H660 | FOXP1-RYBP | Hit Rate | 30ng  (10% input) | 85 | 100% (20/20) |
| K562 | NUP214-XKR3 | Hit Rate | 15ng  (5% input) | 36 | 100% (20/20) |
| Reh | RUNX1-PRDM7 | Hit Rate | 30ng  (10% input) | 84 | 100% (20/20) |

Minimum RNA input was determined as the lowest RNA input which achieved at least 95% hit rate for each fusion. LoD was reported as the mean supporting reads at minimum RNA input for each fusion. Five fusions with ‘*’ were known fusions in selected fusion positive cell lines. The rest three were other fusions detected in fusion positive cell lines.
